# Taxonomic identification of Lower Pleistocene fossil hominins based on distal humeral diaphyseal cross-sectional shape

**DOI:** 10.7717/peerj.1084

**Published:** 2015-07-07

**Authors:** Michael R. Lague

**Affiliations:** Biology Program, Stockton University, Galloway, NJ, USA

**Keywords:** Humerus, Diaphysis, *Paranthropus*, Early *Homo*, Geometric morphometrics, Postcranial skeleton, Taxonomy

## Abstract

The coexistence of multiple hominin species during the Lower Pleistocene has long presented a challenge for taxonomic attribution of isolated postcrania. Although fossil humeri are well-suited for studies of hominin postcranial variation due to their relative abundance, humeral articular morphology has thus far been of limited value for differentiating *Paranthropus* from *Homo*. On the other hand, distal humeral diaphyseal shape has been used to justify such generic distinctions at Swartkrans. The potential utility of humeral diaphyseal shape merits larger-scale quantitative analysis, particularly as it permits the inclusion of fragmentary specimens lacking articular morphology. This study analyzes shape variation of the distal humeral diaphysis among fossil hominins (c. 2-1 Ma) to test the hypothesis that specimens can be divided into distinct morphotypes. Coordinate landmarks were placed on 3D laser scans to quantify cross-sectional shape at a standardized location of the humeral diaphysis (proximal to the olecranon fossa) for a variety of fossil hominins and extant hominids. The fossil sample includes specimens attributed to species based on associated craniodental remains. Mantel tests of matrix correlation were used to assess hypotheses about morphometric relationships among the fossils by comparing empirically-derived Procrustes distance matrices to hypothetical model matrices. Diaphyseal shape variation is consistent with the hypothesis of three distinct morphotypes (*Paranthropus*, *Homo erectus*, non-*erectus* early *Homo*) in both eastern and southern Africa during the observed time period. Specimens attributed to non-*erectus* early *Homo* are unique among hominids with respect to the degree of relative anteroposterior flattening, while *H. erectus* humeri exhibit morphology more similar to that of modern humans. In both geographic regions, *Paranthropus* is characterized by a morphology that is intermediate with respect to those morphological features that differentiate the two forms of early *Homo*. This study demonstrates the utility of the humeral diaphysis for taxonomic identification of isolated postcranial remains and further documents a high degree of postcranial diversity in early *Homo*.

## Introduction

Our ability to reconstruct hominin evolution is predicated upon appropriate taxonomic allocation of fossil specimens. Unfortunately, the diversity of Lower Pleistocene hominins confounds our ability to identify isolated postcrania to species (or even genus), particularly as taxonomic diagnoses are typically based on craniodental remains. While some of the postcranial remains from Swartkrans, South Africa, indicate two distinct morphotypes (presumably representing *Homo* and *Paranthropus*; Grine & Susman, 1991; Susman, de Ruiter & Brain, 2001), taxonomic allocation of isolated bones is, in most cases, controversial and/or uncertain (Grine, 2005). In eastern Africa, the situation is complicated by the existence of multiple contemporaneous forms of early *Homo* (Leakey et al., 2012; Joordens et al., 2013; Antón, Potts & Aiello, 2014; Spoor et al., 2015). Moreover, at least one form of non-*erectus* early *Homo* exhibits aspects of “*Australopithecus*-like” postcranial morphology that had traditionally been assumed to characterize *Paranthropus* (Wood & Constantino, 2007). Hence, isolated postcrania cannot be securely referred to *P. boisei* based solely on their morphological similarity to homologous elements among other australopiths.

Historically, the absence of well-authenticated *P. boisei* skeletons has been a particular problem with respect to identification of eastern African postcranial remains. Until recently, the only skeleton that could reasonably be attributed to *P. boisei* (based on a mandibular fragment) was KNM-ER 1500, a poorly preserved and fragmentary partial skeleton from Koobi Fora (Upper Burgi Member). Although ER 1500 was originally attributed to an australopith (Leakey, 1973) and later more specifically to *P. boisei* (Grausz et al., 1988), Wood (1991) noted that its mandibular morphology can be observed on mandibles attributed to early *Homo*, thereby putting its taxonomic status into question. Fortunately, the recent discovery of a dentally associated partial skeleton (OH 80) more confidently attributed to *P. boisei* (Domínguez-Rodrigo et al., 2013) now provides a stronger foundation for testing taxonomic hypotheses regarding isolated Lower Pleistocene postcrania. Although the skeleton of OH 80 is meager and fragmentary, those remains that are available (portions of a humerus, radius, femur, and tibia) may be compared with homologous specimens from other partial skeletons, as well as with more enigmatic isolated remains.

The humerus, particularly the distal end, is one of the most common postcranial elements in the hominin fossil record, making it ideal for studies of early hominin postcranial variation (e.g., McHenry, 1971; McHenry & Corruccini, 1975; Senut, 1981b; Feldesman, 1982; Lague & Jungers, 1996; Bacon, 2000; McHenry & Brown, 2008; Lague, 2014). Although the new *P. boisei* humerus (OH-80-10) lacks articular morphology, the cross-sectional shape of its distal diaphysis is reportedly similar to isolated specimens from Swartkrans attributed to *P. robustus* (Domínguez-Rodrigo et al., 2013). Based on qualitative observations of transverse sections through the distal diaphysis, Susman, de Ruiter & Brain (2001) attributed humeri with anteroposteriorly flat (mediolaterally broad) distal diaphyses to *P. robustus* (i.e., SKX 19495, SK 24600, SK 2598) and humeri with more “rounded” cross-sections to *Homo* (i.e., SKX 10924, SKX 34805). These attributions were based partly on observations of eastern Africa humeri (Senut, 1981a; see below) and partly on reported (though unsubstantiated^1^1Susman, de Ruiter & Brain (2001) did not provide any metric comparisons, nor include an image of TM 1517 for comparison with the published CT sections of the Swartkrans specimens.) comparisons to TM 1517e, a humerus securely attributed to *P. robustus* (since TM 1517 is the holotype of that taxon; Broom, 1938; Broom & Schepers, 1946).

The above taxonomic attributions at Swartkrans are less than secure, particularly in the absence of any quantitative comparison with TM 1517 (or other available fossil humeri). In addition, recent evidence from the distal articular surface suggests that SK 24600 and SKX 10924 (the only two specimens from Swartkrans to include elbow joint morphology) actually belong to *Homo* and *Paranthropus*, respectively (Lague, 2014; *contra* Susman, de Ruiter & Brain, 2001). Hence, there is some question as to whether those specimens at Swartkrans characterized by AP flattening are truly representative of *P. robustus* rather than *Homo*.

Given the AP flattened diaphyseal morphology of OH 80-10, the dichotomous pattern of shape variation observed at Swartkrans may also characterize fossils in eastern Africa of similar age. In fact, KNM-ER 739, an isolated specimen tentatively attributed to *Australopithecus* (and therefore, by inference, *P. boisei*; Leakey, 1971) is described as having an unusually AP flattened distal diaphysis compared to Gomboré IB 7594, a specimen from Melka Kunturé, Ethiopia, commonly attributed to *Homo* (Chavaillon et al., 1977; Senut, 1981a). These two specimens are also considerably different in distal articular morphology (Lague & Jungers, 1996), supporting the contention that they do, in fact, represent different taxa. Unfortunately, neither of these specimens is associated with taxonomically diagnostic craniodental material and therefore cannot, on their own, resolve questions regarding which genus (if either) has characteristically AP flattened distal humeri. Moreover, other than the description of OH-80 (Domínguez-Rodrigo et al., 2013), no study has assessed distal diaphyseal shape among available eastern African humeral specimens of known species attribution (i.e., OH 62, KNM-ER 3735, ER 1808, WT 15000).

The potential taxonomic utility of humeral diaphyseal shape merits quantitative analysis of a wide range of hominin fossils from across Africa. This study analyzes cross-sectional shape variation of the distal humeral diaphysis among fossil hominins (c. 2-1 Ma) attributable to *Paranthropus* and *Homo* to test the hypothesis that specimens can be divided into distinct morphotypes. Fossils from both southern and eastern Africa, including fragmentary specimens lacking articular morphology, are examined to assess whether a similar pattern of morphological variation characterizes each region.

## Materials and Methods

The main fossil study group includes 15 hominin humeri (c. 2-1 Ma), six of which are attributed to species based on association with craniodental material (Table 1). In most cases, 3D surface scans of the original specimens (collected by JM Plavcan and CV Ward) were used to find the transverse section of interest (see below). The scan of OH 62 was produced from a high-quality cast housed at the Institute of Human Origins (Tempe, AZ). An image of the appropriate section of OH 80-10 (as determined by the author) was kindly provided by M Domínguez-Rodrigo from a CT scan. In the absence of available 3D scans of SKX 19495 and SK 2598, I used the published CT sections of those specimens (Susman, de Ruiter & Brain, 2001). Two other published CT sections from the same source (SK 24600, SKX 10924) were used to assess the effect of using published images rather than sections collected by the author (from 3D surface scans). Shape differences between the two sets of data were found to be negligible; for both specimens, the shape provided in the literature was far more similar to the shape collected from the surface scan than to any other specimen (based on Procrustes distances; see below). Since this study relies on Procrustes distance matrices to represent morphometric relationships among the fossils, it is desirable that different sections representing the same specimen should produce very similar sets of Procrustes distances (to the other fossils). In the case of both SK 24600 and SKX 10924, there is, in fact, a significant correlation between the two sets of Procrustes distances (SKX 10924: *R* = 0.85, *P* < 0.001; SK 24600: *R* = 0.85, *P* < 0.001), suggesting that the use of published scans for SK 24600 and SKX 10924 will not significantly impact the results.

**Table 1:** Main study group of fossil hominin humeri.

	Specimen	Bed/Member	Taxon^a^	Reference(s)^b^
	**South Africa** ^c^			
1	TM 1517	Mb. 3 (Kromdraai)	***P. robustus***	Broom (1938); Straus Jr (1948)
2	SKX 10924	Member 3	?? *Homo*	Susman, de Ruiter & Brain (2001)
3	SKX 19495	Member 3	?? *P. robustus*	Susman, de Ruiter & Brain (2001)
4	SK 2598	Member 1 (HR)	?? *P. robustus*	Susman, de Ruiter & Brain (2001)
5	SKX 34805	Member 1 (LB)	?? *Homo*	Susman (1989); Susman, de Ruiter & Brain (2001)
6	SK 24600	Member 1 (LB)	?? *P. robustus*	Susman, de Ruiter & Brain (2001)
	**Eastern Africa**			
7	OH 80	Bed II (Olduvai)	***P. boisei***	Domínguez-Rodrigo et al. (2013)
8	OH 62	Bed I (Oludvai)	***Homo habilis***	Johanson et al. (1987)
9	KNM-WT 15000	Natoo	***Homo ergaster***	Walker & Leakey (1993)
10	KNM-ER 739	Okote	?? *P. boisei*	Leakey, Mungai & Walker (1972); McHenry (1973)
11	KNM-ER 1591	KBS	?? *Homo*	Leakey (1973)
12	KNM-ER 1808	KBS	***Homo ergaster***	Leakey (1974); Leakey & Walker (1985)
13	KNM-ER 6020	KBS	?? *P. boisei*	Leakey & Walker (1985)
14	KNM-ER 1504	Upper Burgi	?? *P. boisei*	Leakey (1973)
15	KNM-ER 3735	Upper Burgi	***Homo habilis***	Leakey & Walker (1985)

**Notes.**

a Taxa in bold indicate specimens associated with taxonomically-identifiable craniodental material.

b Paper(s) in which the given specimen was originally published, described, and/or attributed to taxon.

c All specimens are from Swartkrans unless otherwise noted.

Some issues associated with certain fossil specimens merit attention. First, although the KNM-ER 1808 (*H. erectus*) skeleton is pathological, periosteal bone deposits are much thinner in the upper limb than in the lower limb (Ruff, 2008; Dolan, 2011). Minor surface irregularities (mostly due to small amounts of missing bone) were ignored when quantifying diaphyseal contour and the pathology is not expected to have a significant effect on overall cross-sectional shape. Second, the subadult KNM-WT 15000 (*H. erectus*) humerus is included here with the understanding that its shape may be partly affected by its developmental status (as demonstrated for its relative cortical thickness; Ruff, 2008). Lastly, although KNM-ER 1591 was originally attributed to *Homo* (Leakey, 1973), it has not been universally accepted as a hominin (Walker & Leakey, 1993). Its inclusion in this study is based on the author’s observation that the size and shape of its diaphysis (most of which is preserved) is so similar to that of KNM-ER 739 (also from Ileret) that the two specimens are likely to be conspecific.

Additional taxa were sampled to provide a comparative context for interpreting observed morphological variation among the fossils of the main study group (Table 2). Scans were collected for four species of *Australopithecus* (eight specimens total), as well as for four extant hominid species (*Homo sapiens*, *Pan troglodytes*, *Gorilla gorilla*, *Pongo pygmaeus*). Cross-sectional shape of AL 288-1 was calculated for both left and right humeral sections.

**Table 2:** Comparative sample.

Species	*N*	Specimens^a^ or Museum Source^b^
*Au. anamensis*	1	KNM-KP-271
*Au. afarensis*	4	AL 137-48a, AL 288-1 (l, r), AL 322-1
*Au. africanus*	1	Stw 431
*Au. sediba*	2	MH1, MH2
*Homo sapiens*	21	CMNH and NMNH
*Pan troglodytes*	21	CMNH
*Gorilla gorilla*	12	CMNH
*Pongo pygmaeus*	11	CMNH and NMNH

**Notes.**

a All scans collected from high-quality casts by the author except for Stw 431. Scan of original specimen of Stw 431 collected by JM Plavcan.

b CMNH, Cleveland Museum of Natural History; NMNH, Smithsonian National Museum of Natural History (Washington, DC). All scans collected by JM Plavcan.

For each 3D scan, *Landmark editor* software (Wiley et al., 2005) was used to define a transverse section through the diaphysis intended to closely match that of Susman, de Ruiter & Brain (2001). All left humeri were mirrored to create “right” humeri for data collection. For each specimen, a user-defined transverse plane was created that could be virtually “slid” in either direction (proximodistally) until placed at the appropriate section level. The section of interest is located just proximal to a shallow depression on the posterior diaphyseal surface that slopes distally toward the much more highly excavated olecranon fossa. Although “percentage of total bone length” represents one possible way of defining the placement of a diaphyseal section, none of the fossils preserve the entire humerus. Consistency of section placement on all specimens was therefore accomplished by using one specimen as a constant visual reference. When located on complete humeri of four different extant hominid genera (*n* = 57), the section of interest typically corresponds to ∼19% of bone length from the most distal point on the lateral trochlear crest $(\bar {x}=19.1,s=1.42)$. The potential effect of variation in section placement on cross-sectional shape was considered via the additional collection of data from four length-defined sections (15%, 17%, 19%, 21%) on every modern human individual.

**Figure 1:**
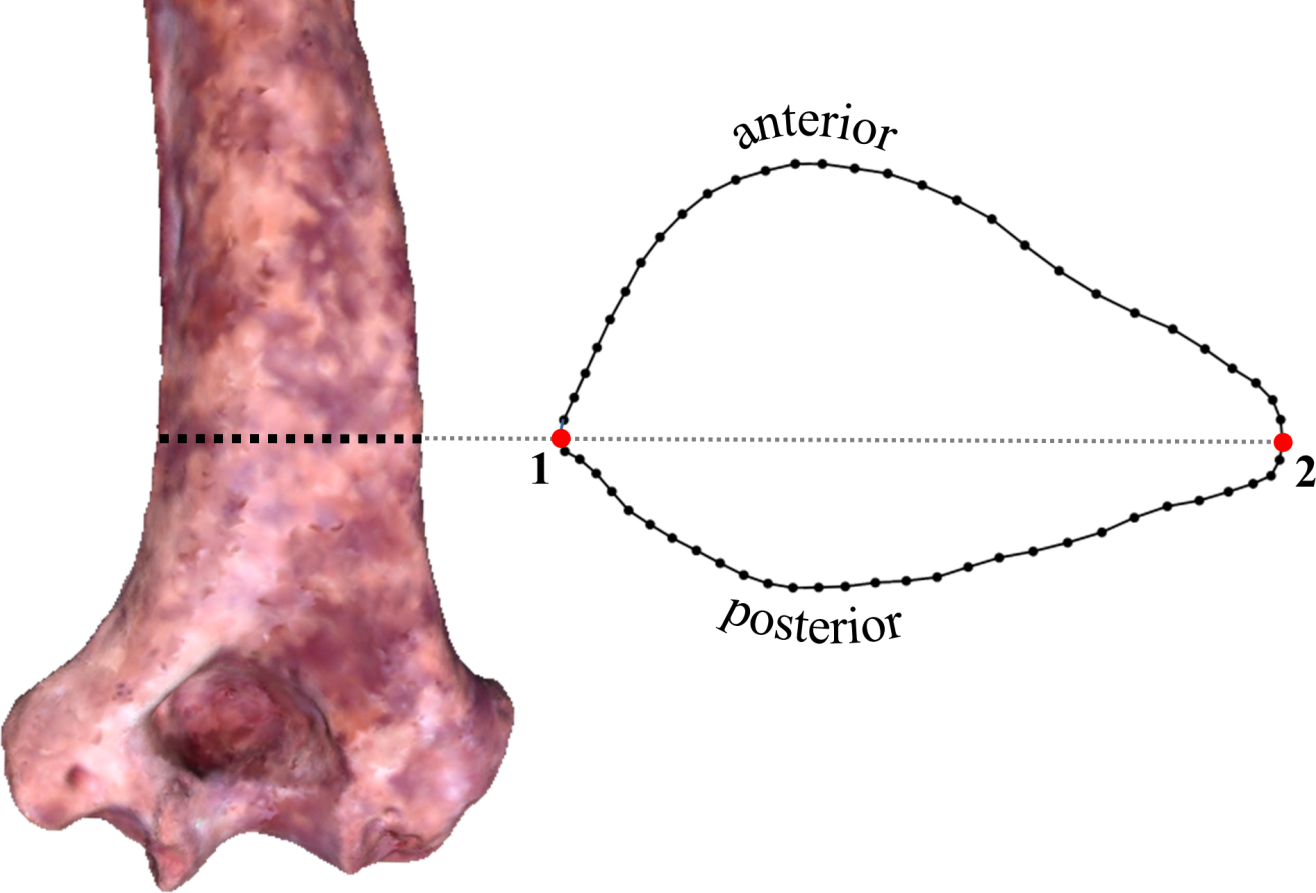
Transverse section and two-dimensional landmarks used in this study. Section location is shown on a 3D surface scan of KNM-ER 739 (posterior view). The two numbered landmarks represent the most medial (1) and lateral (2) points on the section. All other points (58) represent sliding semilandmarks.

Cross-sectional shape was initially captured by placing a high density of three-dimensional (3D) landmarks around the bone surface along the appropriately positioned transverse plane (via the “curve” option in *Landmark editor*). For each individual specimen, the 3D landmark coordinates were then exported from *Landmark editor* and converted to 2D landmarks by mathematically aligning the landmark configuration to its principal axes, thereby virtually eliminating variation along a third dimension. Graphs of the resulting 2D surface outlines were imported into *tpsDig2* (Rohlf, 2013) for quantification of 60 two-dimensional coordinate points, including two Type 2 landmarks (i.e., the most medial and lateral points of the section) and a set of 29 sliding semilandmarks along both the anterior and posterior surfaces (Fig. 1; see definitions in Zelditch, Swiderski & Sheets, 2012). For specimens suffering from minor surface abrasions, missing material was easily accounted for by using adjacent surface contours to bridge across obvious irregularities in the outline. Unfortunately, the appropriate section of OH 62 is missing substantial amounts of material on both its medial and lateral aspects and is estimated to be only about 63% complete. Therefore, the OH 62 section was digitally reconstructed based on its available contours and comparisons with all of the other specimens.^2^2It is worth noting that the preserved surface contours of the OH 62 section are particularly similar to those of KNM-ER 3735, which reportedly belongs to the same taxon (Antón, Potts & Aiello, 2014). Since the accuracy of this reconstruction is unknown, the tests described below were performed for two sets of specimens differing only in the presence/absence of OH 62.

Generalized orthogonal least-squares Procrustes analysis (GPA) was used to transform raw landmark configurations into the same shape space (i.e., to superimpose shapes; Gower, 1975; Rohlf & Slice, 1990). With the use of *tpsRelw* software (Rohlf, 2010), an iterative procedure was used in which semilandmarks (designated via a ‘sliders’ file) were allowed to slide along surface outlines to minimize the amount of shape change between each specimen and the Procrustes average of all specimens. Procrustes distances (*D_p_*) were calculated between every pair of fossils as a measure of shape dissimilarity (corresponding to the square root of the sum of squared distances between corresponding landmarks of two superimposed shapes; Rohlf, 1999; Zelditch, Swiderski & Sheets, 2012). To provide a basis for evaluating Procrustes distances between fossils, an exact randomization procedure (Sokal & Rohlf, 1981) was used in which all possible pairwise Procrustes distances were calculated within each extant species. The magnitude of a given Procrustes distance between fossils could then be evaluated by counting the number of pairwise distances in the extant reference group with a higher value.

Principal Components Analysis (PCA; equivalent herein to *relative warps analysis*) was used to visually summarize variation among the superimposed shapes. Although distance-based cluster analysis (UPGMA; Unweighted Pair Group Method with Arithmetic Mean) was also explored as a means of visualization, it was generally too sensitive to distortions caused by dimensional reduction (thereby yielding insufficiently high cophenetic correlations). Only cluster analyses yielding a cophenetic correlation higher than 0.8 were considered reasonably good representations of the Procrustes distance matrix upon which they were based.

**Figure 2:**
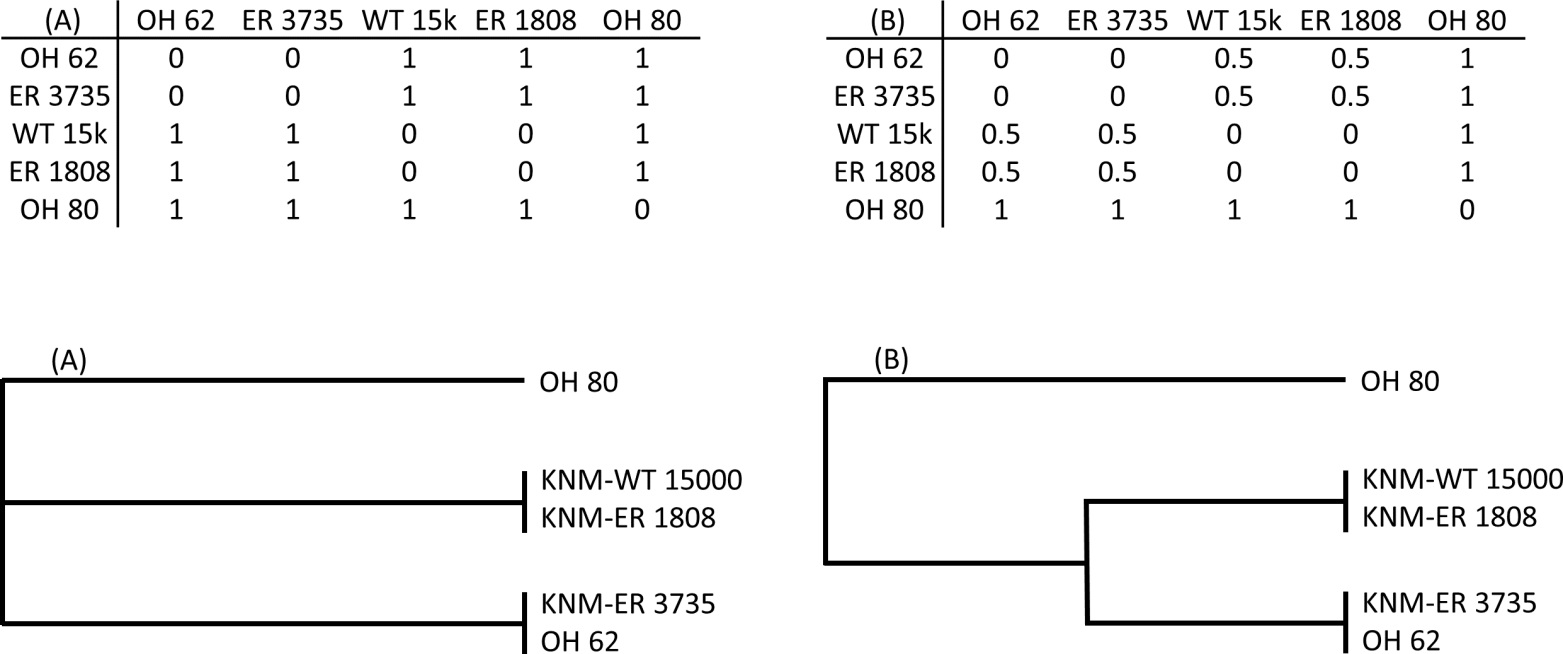
Two hypothetical examples (A, B) of model matrices (for a subset of five fossils). For each matrix, a dendrogram is shown that depicts the relationships defined therein. Matrix (A) represents a hypothesis of three independent groups with respect to the morphological relationships among specimens. Matrix (B) is similar except that members of two of the groups are hypothesized to be more similar to one another than they are to OH 80.

To explicitly test hypotheses regarding taxonomic relationships among the fossils, I used Mantel tests of matrix correlation (Mantel, 1967; Rodrigues et al., 2002). All tests were performed with 5,000 random permutations using PAST software (Hammer, Harper & Ryan, 2001). The null hypothesis of each test is that there is no relationship between the structure of two matrices under consideration: (1) the observed Procrustes distance (*D_p_*) matrix for the fossils, and (2) a model matrix consisting of user-assigned hypothetical distance values (Fig. 2). For each pair of specimens in a given model matrix, a discrete distance value was assigned to indicate hypothesized membership in the same taxon (0) or a different taxon (1). In some cases, an intermediate value (0.5) was used to indicate different species of the same genus (e.g., two different *Paranthropus* species, or two different *Homo* species). The patterns of linkages established in the model matrices were used to test various hypothesis regarding the morphological relationships among the fossils (as represented by the observed matrix of Procrustes distances).

It is beyond the scope of this paper to test every possible way of dividing 15 fossils into multiple groups. Group configurations tested here are based on previously published taxonomic hypotheses and/or the results of the tests themselves (i.e., arrangement on PC plots and/or observed Procrustes distance values). For any given Mantel test, rejection of the null hypothesis does not necessarily imply that the group configuration under testing reflects the “best” or “true” taxonomic grouping, since multiple group configurations may yield significant results. The Mantel tests are meant only to refute or support a given taxonomic hypothesis.

## Results

### South Africa

**Figure 3:**
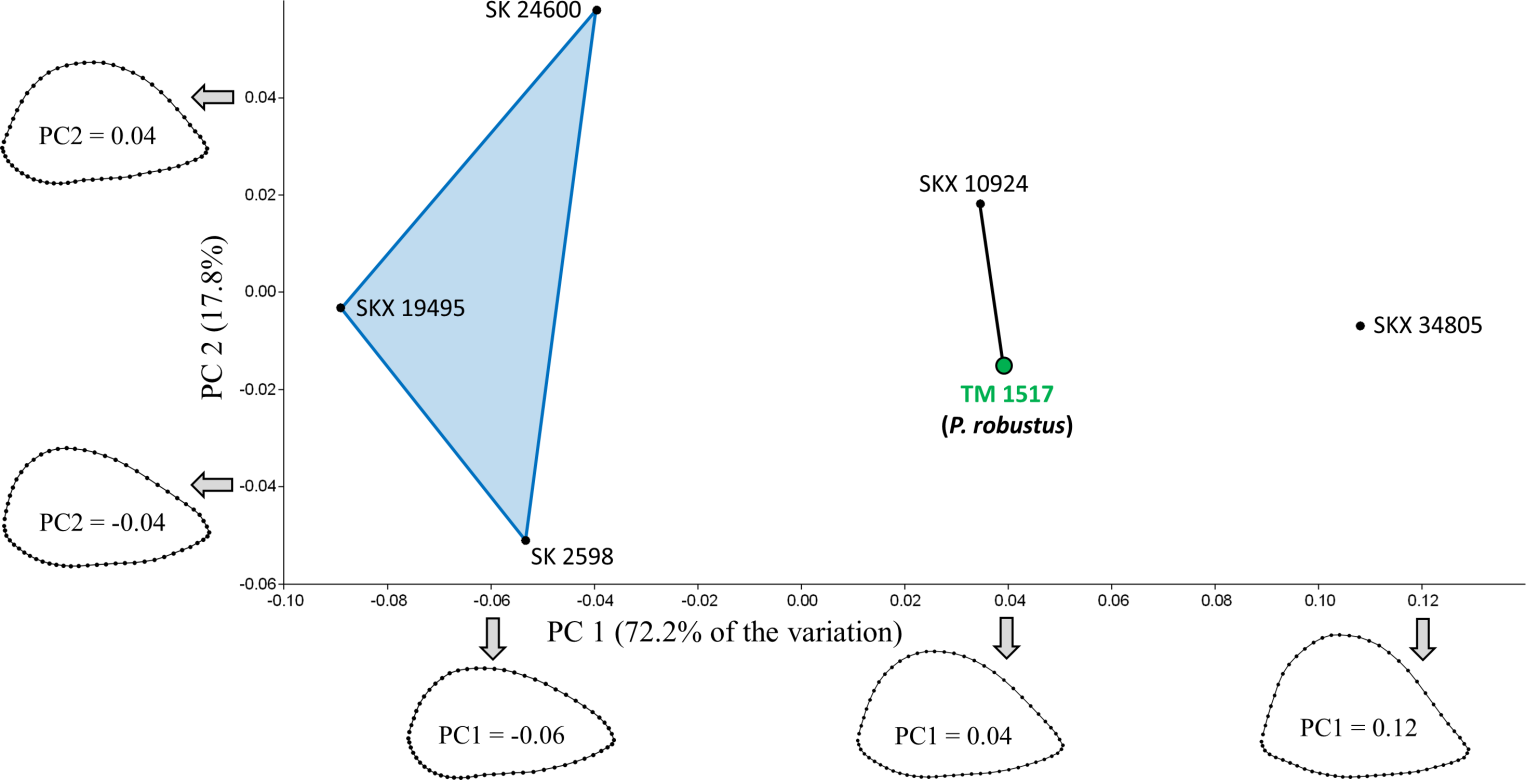
PCA results for the South African fossil study group. Only TM 1517 is associated with taxonomically identifiable craniodental remains. Shape variation along PC1 largely involves differences in AP width relative to ML width. Results of the Mantel tests of matrix correlation support the three-group configuration depicted on the plot. SKX 10924 is morphologically very similar to *P. robustus*.

PCA of the South African specimens reveals that most of the shape variation (72.2%) occurs along a single principal component (PC1; Fig. 3). PC1 is associated with variation in relative AP width and reflects the variation described by Susman, de Ruiter & Brain (2001) such that three AP flattened specimens (SK 24600, SKX 19495, SK 2598) are separated from two more rounded specimens (SKX 10924, SK 34805). Contrary to the claims of Susman and colleagues, however, TM 1517 (*P. robustus*) is more similar to the rounded specimens than to the AP flattened specimens; in fact, the Procrustes distance (0.057) between TM 1517 and SK 10924 is lower than that for any other pair of South African specimens (Table 3). If one also considers South African fossils *outside* of the main study group, only the two Malapa specimens (MH1, MH2) have a smaller *D_p_* between them.

**Table 3:** Pairwise Procrustes distances among hominin fossils (lower diagonal) and results of exact randomization (upper diagonal).^a^^,^^b^

	***P. robustus***		**Non-*erectus* early *Homo***					***H. erectus***			***P. boisei***					***Australopithecus***							
Fossil specimen	TM 1517	SKX 10924	SKX 19495	SK 2598	SK 24600	OH 62	KNM-ER 3735	SKX 34805	KNM-WT 15000	KNM-ER 1808	OH 80	KNM-ER 739	KNM-ER 1591	KNM-ER 6020	KNM-ER 1504	KNM-KP 271	AL 288-1m	AL 288-1s	AL 137-48a	AL 322-1	Stw 431	MH1	MH2
TM 1517		–	P	–	–	–	–	–	–	–	–	–	–	–	–	–	–	–	–	–	–	–	–
SKX 10924	0.057		P	–	–	–	–	–	–	–	–	–	–	–	–	–	–	–	–	–	–	–	–
SKX 19495	**0.136**	**0.134**		–	–	–	–	PHGO	PHGO	PHGO	–	–	–	P	–	PHG	P	PHGO	–	PHGO	PHG	PHG	PHG
SK 2598	0.102	0.117	0.069		–	–	–	PHGO	PHGO	P	–	–	–	–	–	–	–	P	–	P	P	–	–
SK 24600	0.110	0.098	0.086	0.111		–	–	PHGO	–	PHG	–	–	–	–	–	PHG	–	PHG	–	PHG	–	P	PHG
OH 62	0.081	0.098	0.099	0.087	0.072		–	PHG	P	–	–	–	–	–	–	–	–	–	–	PHG	–	–	–
KNM-ER 3735	0.103	0.107	0.101	0.111	0.045	0.047		PHG	P	PHG	–	–	–	–	–	PHG	–	PHG	–	PHG	–	–	PH
SKX 34805	0.084	0.097	**0.198**	**0.171**	**0.164**	**0.149**	**0.161**		–	–	PHG	–	–	PH	–	–	–	–	–	–	–	–	PHG
KNM-WT 15000	0.082	0.072	**0.180**	**0.164**	0.129	**0.132**	**0.132**	0.067		–	–	–	–	–	–	–	–	–	–	–	–	P	PHG
KNM-ER 1808	0.078	0.088	**0.172**	**0.132**	**0.151**	0.126	**0.150**	0.091	0.106		–	–	–	–	–	–	–	–	–	–	–	–	–
OH 80	0.086	0.068	0.089	0.076	0.080	0.086	0.096	**0.144**	0.118	0.106		–	–	–	–	–	–	–	–	–	–	–	–
KNM-ER 739	0.062	0.082	0.108	0.067	0.109	0.081	0.106	0.120	0.120	0.085	0.069		–	–	–	–	–	–	–	–	–	–	–
KNM-ER 1591	0.055	0.063	0.117	0.081	0.107	0.090	0.111	0.111	0.104	0.073	0.058	0.044		–	–	–	–	–	–	–	–	–	–
KNM-ER 6020	0.101	0.056	**0.135**	0.128	0.106	0.118	0.123	**0.139**	0.111	0.110	0.067	0.100	0.090		–	–	–	–	–	–	–	–	P
KNM-ER 1504	0.069	0.057	0.117	0.109	0.073	0.067	0.072	0.118	0.088	0.106	0.068	0.075	0.077	0.072		–	–	–	–	–	–	–	–
KNM-KP-271	0.086	0.077	**0.145**	0.113	**0.148**	0.127	**0.154**	0.122	0.128	0.089	0.090	0.080	0.073	0.086	0.107		–	–	–	–	–	–	–
AL 288-1m	0.073	0.057	**0.134**	0.111	0.107	0.092	0.113	0.119	0.097	0.076	0.064	0.080	0.066	0.065	0.066	0.075		–	–	–	–	–	–
AL 288-1s	0.073	0.069	**0.165**	0.131	**0.141**	0.123	**0.146**	0.094	0.088	0.052	0.093	0.091	0.069	0.094	0.096	0.073	0.050		–	–	–	–	–
AL 137-48a	0.053	0.061	0.126	0.088	0.118	0.097	0.122	0.100	0.100	0.059	0.065	0.043	0.034	0.084	0.075	0.059	0.055	0.053		–	–	–	–
AL 322-1	0.082	0.057	**0.168**	**0.139**	**0.149**	**0.141**	**0.158**	0.100	0.091	0.087	0.096	0.101	0.080	0.078	0.101	0.062	0.075	0.059	0.067		–	–	–
Stw 431	0.071	0.067	**0.158**	**0.139**	0.115	0.122	0.122	0.086	0.055	0.096	0.097	0.106	0.086	0.104	0.089	0.120	0.092	0.085	0.086	0.089		–	P
MH1	0.074	0.101	**0.142**	0.100	**0.131**	0.084	0.118	0.122	**0.130**	0.071	0.099	0.057	0.076	0.117	0.089	0.096	0.080	0.089	0.066	0.119	0.121		–
MH2	0.093	0.119	**0.153**	0.100	**0.152**	0.103	**0.139**	**0.149**	**0.154**	0.083	0.110	0.078	0.089	**0.131**	0.114	0.106	0.093	0.099	0.081	0.128	**0.135**	0.048	
*H. sapiens*	0.054	0.059	0.166	0.139	0.125	0.109	0.124	0.072	0.053	0.063	0.097	0.094	0.076	0.095	0.076	0.099	0.059	0.046	0.066	0.074	0.061	0.089	0.109
*P. troglodytes*	0.077	0.063	0.150	0.124	0.134	0.127	0.146	0.096	0.094	0.063	0.082	0.087	0.065	0.082	0.096	0.060	0.060	0.043	0.052	0.057	0.089	0.100	0.116
*G. gorilla*	0.095	0.052	0.148	0.144	0.093	0.108	0.106	0.126	0.087	0.108	0.081	0.110	0.094	0.048	0.064	0.109	0.065	0.093	0.094	0.090	0.088	0.118	0.138
*P. pygmaeus*	0.061	0.052	0.161	0.140	0.114	0.100	0.112	0.089	0.077	0.072	0.096	0.092	0.081	0.078	0.069	0.096	0.064	0.072	0.076	0.084	0.080	0.087	0.110

**Notes.**

a Fossils specimens are arranged into taxa based on the results presented herein. Fossils outside the main study group are shaded in gray. Procrustes distances to extant species averages are also provided.

b Procrustes distances in bold are those found to be higher than 95% of intraspecific pairwise Procrutes distances in at least one extant reference species. Letters above the diagonal indicate the particular species (if any) for which the given fossil distance was found to be significantly high, labeled as follows: P, *Pan*; H, *Homo*; G, *Gorilla*; O, *Pongo*.

Of the two model matrices in which the six specimens were divided into two groups (Tests #1 and #2; Table 4), neither yields a significantly high correlation coefficient with the observed distance matrix, regardless of whether TM 1517 is linked to the three AP flattened specimens (c.f., Susman, de Ruiter & Brain, 2001; *R* = .371, *P* = 0.113) or with the two more rounded specimens (*R* = .677, *P* = 0.075). Of the three tests (#3–5) involving division of specimens into three groups, only one (Test #3) results in rejection of the null hypothesis (*R* = .550, *P* = 0.034). Hence, among those tested here, the model matrix that best reflects the observed distances (*D_p_*) among specimens is one defined by the following three groups: (1) SK 24600, SKX 19495, SK 2598; (2) TM 1517, SKX 10924; (3) SKX 34805. All three *D_p_* values between SKX 34805 and the members of the first group exceed the majority (>95%) of pairwise distances sampled within all four extant species (Table 3). The two members of the second group, being morphologically intermediate between members of the other two groups, are only unusually distant from SKX 19495 (and only relative to variation in *P. troglodytes*).

**Table 4:** Results of Mantel tests of matrix correlation.^a^

		Test set:	South Africa only					Eastern Africa only					Mixed regions				
		Test #:	1	2	3	4	5	6	7	8	9	10	11	12	13	14	15
Knowns	TM 1517		1	1	1	1	1							1	1	1a	1
	OH 80							1	1	1	1	1		2	2	1b	2
	KNM-ER 3735/OH 62							2	2	2	2	2a	1	2	3	2	3a
	KNM-WT 15000/ER 1808							3	3	3	3	2b		2	4	3	3b
Unknowns	SKX 10924		2	1	1	3	3							1	1	1a	1
	SK(X) 2598/19495/24600		1	2	2	2	2						2	1	3	2	3a
	SKW 34805		2	1	3	1	3							1	4	3	3b
	ER 739/1504/6020/1591							2	3	4	1	1		2	2	1b	2
	Results w/o OH 62	*R*	0.371	0.677	0.550	0.438	0.397	0.161	0.166	0.315	0.621	0.490		-0.005	0.380	0.512	0.097
		*P*	0.113	0.075	0.034	0.100	0.107	0.262	0.305	0.084	0.004	0.007		0.443	0.0004	0.0002	0.156
			ns	ns	*	ns	ns	ns	ns	ns	**	**		ns	***	***	ns
	Results with OH 62	*R*						0.279	0.216	0.375	0.598	0.415	0.151	0.008	0.392	0.504	0.104
		*P*						0.114	0.153	0.016	0.001	0.002	0.311	0.396	0.0002	0.0002	0.153
								ns	ns	*	**	**	ns	ns	***	***	ns

**Notes.**

a Specimens listed with same number were considered members of the same group for purposes of the given Mantel test. The particular number designations in the table are arbitrary and meant only to convey information on group membership being tested. Groups with different letters (a, b) for the same number were tested as “linked” (i.e., different species of the same genus).

Test results indicated as follows ns not significant * *P* < 0.05 ** *P* < 0.01 *** *P* < 0.001

### Eastern Africa

In the eastern African sample, over half of the specimens (five of nine) are attributed to species based on associated craniodental material. The remaining four specimens (referred to as “unknowns” below) derive from Koobi Fora.

**Figure 4:**
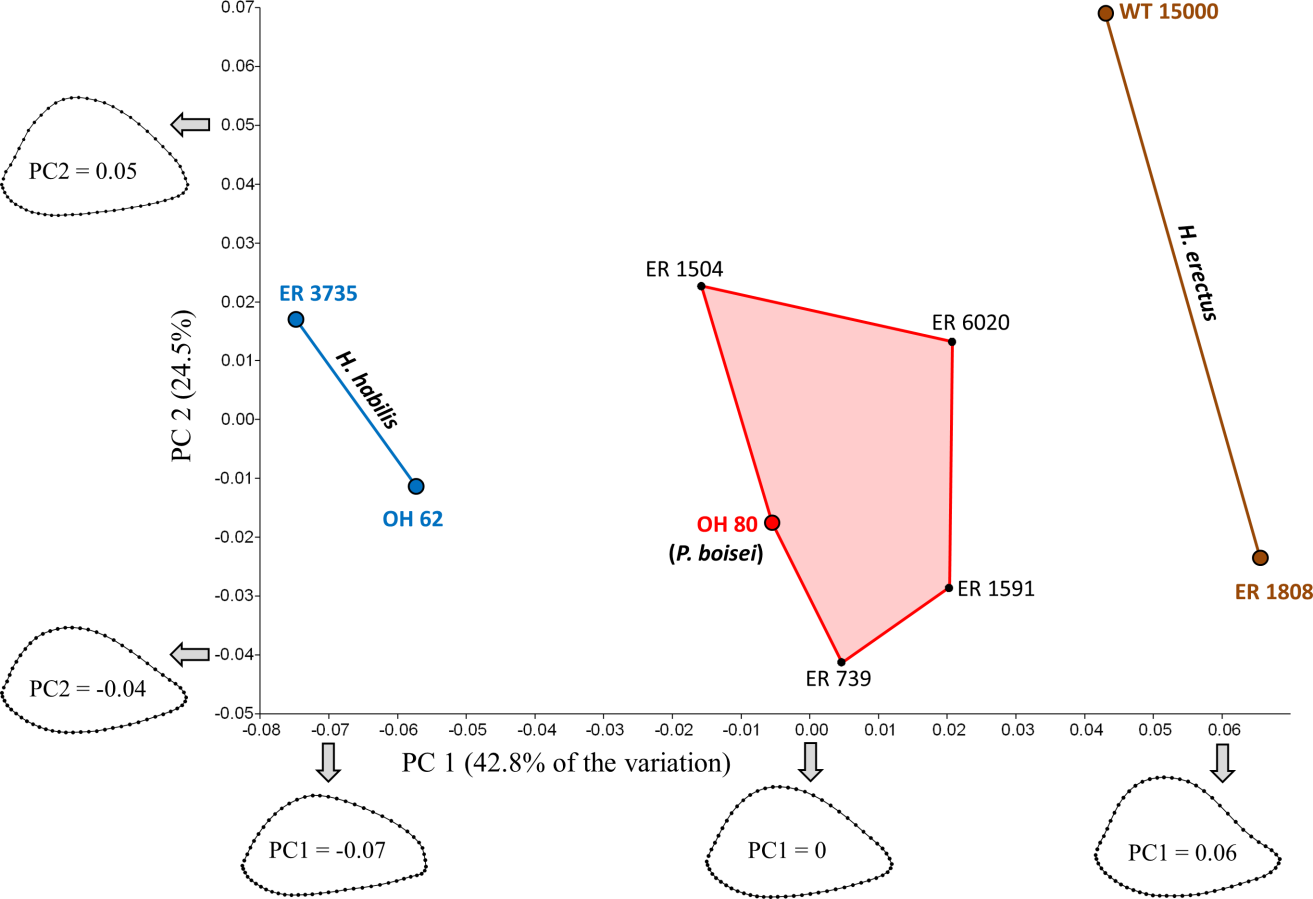
PCA results for the eastern African fossil study group. Specimens associated with taxonomically identifiable craniodental remains are indicated with larger colored dots. As in South Africa, shape variation along PC1 involves differences in AP width relative to ML width. Results of the Mantel tests support the three-group configuration depicted on the plot. The four “unknown” Koobi Fora specimens likely represent *P. boisei*.

The first two principal components account for 67.3% of the variation (Fig. 4). Shape variation along PC1 is remarkably similar to that observed for South Africa, such that specimens with low scores (in this case, those attributed to *H. habilis*) have more AP flattened cross sections, while those with high scores (i.e., those attributed to *H. erectus*) have more rounded sections. Shape differences between the two *Homo* groups are also apparent on the anterolateral surface, which is slightly concave in *H. erectus* and more convex in *H. habilis*; the same pattern of shape variation is also observed (to a lesser extent) along PC1 of the South African PCA. Along PC1, all four of the Koobi Fora “unknowns” cluster together in the middle, in close proximity to OH 80 (*P. boisei*). Based on Procrustes distances (Table 3), among the eastern African specimens, two of the “unknowns” (ER 1504, ER 6020) are morphologically more similar to OH 80 than to any other specimen,^3^3The Procrustes distance between KNM-ER 1504 and OH 62 (0.067) is actually slightly smaller than that between ER 1504 and OH 80 (0.068). As noted, however, OH 62 required considerable reconstruction and its associated distance values should be treated with greater caution. whilst ER 739 and ER 1591 are closer only to each other. In fact, the Procrustes distance (0.044) between ER 739 and ER 1591 is smaller than that for any other pair of fossils in the eastern African sample.

The shape similarity among the “unknowns” suggests coding them as members of the same taxon for purposes of the Mantel tests. In fact, previous studies of distal articular morphology also show strong shape similarity among the unallocated Koobi Fora humeri (Lague & Jungers, 1996; Lague, 2014; but see McHenry & Brown, 2008). As the specimens appear to comprise a morphologically cohesive group, and there is little basis for separating them, all of the “unknowns” were considered members of the same taxon for purposes of the eastern African Mantel tests.

The similarity of the “unknowns” to OH 80 is further supported by the Mantel test results (Table 4). With one exception, results are the same whether or not one includes the reconstructed OH 62 humerus; unless otherwise noted, values reported below are from tests in which OH 62 was excluded. Model matrices in which the Koobi Fora “unknowns” were coded as *H. habilis* (Test #6: *R* = 0.161, *P* = 0.262) or *H. erectus* (Test #7: *R* = 0.166, *P* = 0.305) are not correlated with the observed Procrustes distance matrix. Moreover, treatment of the “unknowns” as their own distinct group (i.e., not linked to any of the three known species) also produces a nonsignificant result (Test #8: *R* = 0.315, *P* = 0.084) unless OH 62 is included in the sample (*R* = 0.375, *P* = 0.016). In contrast, treatment of the four “unknowns” as *P. boisei* (i.e., grouping them with OH 80) yields a considerably higher and statistically significant correlation (Test #9: *R* = 0.621, *P* = 0.004).

A model matrix in which the two *Homo* species were linked (by assigning 0.5 for pairings between different species of *Homo*) also results in rejection of the null hypothesis (Test #10: *R* = 0.490, *P* = 0.007). Nonetheless, the correlation between observed and model matrices is much higher when the two *Homo* groups are treated independently, as one would expect given that the two species of *Homo* fall on opposite extremes of PC1. Moreover, three of the four possible interspecific Procrustes distances between *H. habilis* and *H. erectus* are unusually high (>95% of all pairwise values; Table 3) in one or more of the extant reference groups, with the distance between ER 1808 (*H. erectus*) and ER 3735 (*H. habilis*) being the largest in the eastern African sample.

### Combined sample

**Figure 5:**
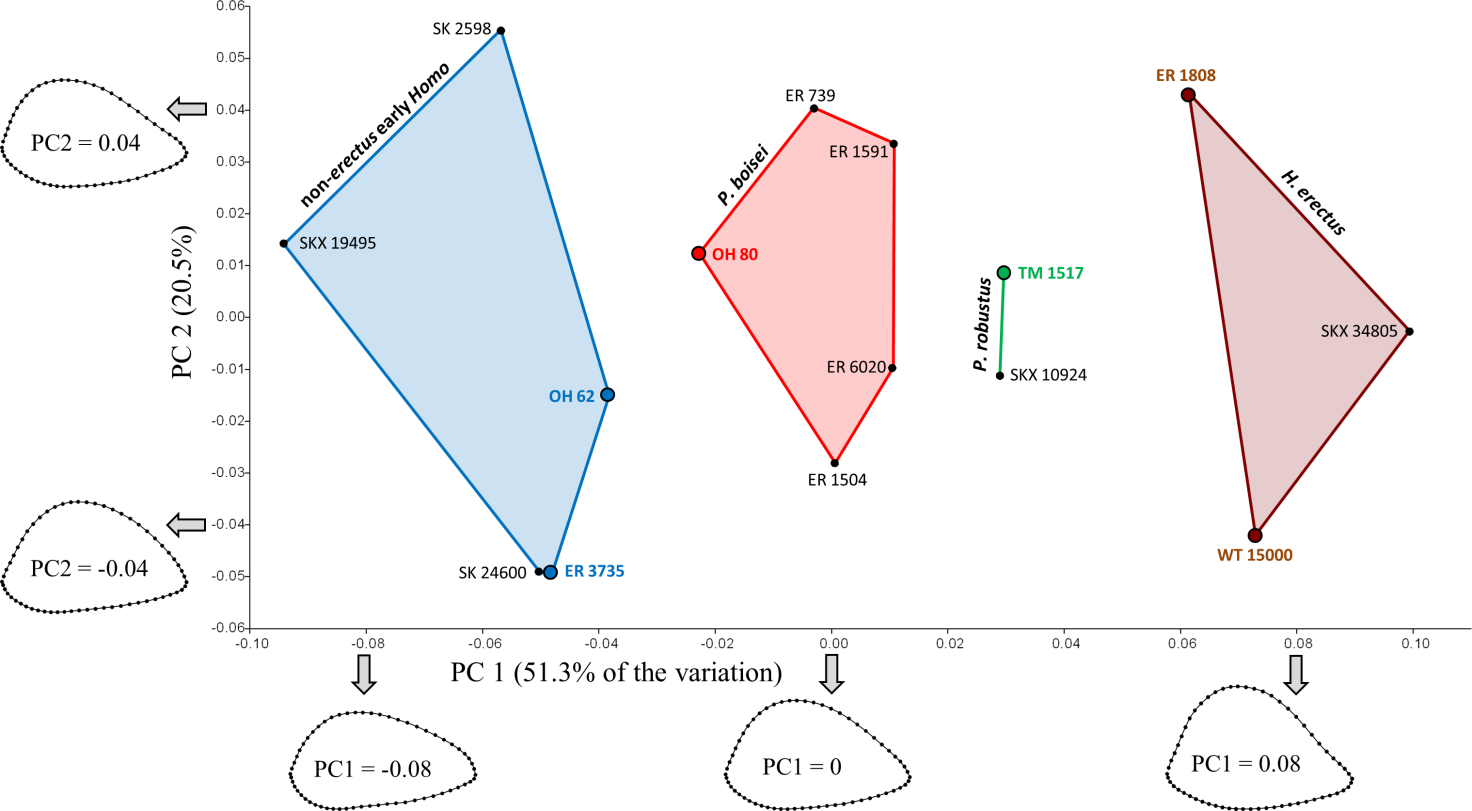
PCA results (PC1, PC2) for the main study group (combined geographic regions). Specimens associated with taxonomically identifiable craniodental remains are indicated with larger colored dots. Shape variation along PC1 is similar to that observed for region-specific PCAs (Figs. 3 and 4). The two species of *Paranthropus* are both intermediate with respect to those morphological features that differentiate *H. erectus* from non-*erectus* early *Homo*.

When all of the fossil specimens are combined, PC1 is again associated with a pattern of shape variation in which AP flatter specimens have lower scores (Fig. 5). The first two principal components account for over 70% of the shape variation in the sample, though most of the between-group variation is associated with PC1. The arrangement of specimens along PC1 resembles a combination of the previous region-specific PCA results. Mantel Test #12 supports the hypothesis that shape-based distances among specimens do not reflect a simple regional dichotomy (i.e., eastern vs. southern; *R* = − 0.005, *P* = 0.443).

Along PC1, SKX 34805 has the highest positive score, thereby falling closest to the two Kenyan specimens attributed to *H. erectus*. At the other extreme, the three Swartkrans specimens originally attributed to *P. robustus* (Susman, de Ruiter & Brain, 2001) are much closer to the two *H. habilis* specimens (OH 62, ER 3735) than to TM 1517 (*P. robustus*). A Mantel test (#11) involving only the two *H. habilis* humeri and the three aforementioned Swartkrans humeri does not support the separation of these specimens into two groups, largely because the Procrustes distance (0.045) between SK 24600 and KNM-ER 3735 is unusually small (i.e., second only to that between ER 739 and ER 1591 among fossils of the main study group).

Based on the results presented above, it is reasonable to consider the following four-group configuration (also see Table 5): (1) non-*erectus* early *Homo* (OH 62, ER 3735, SK 24600, SKX 19495, SK 2598), (2) *P. boisei* (OH 80, ER 739, 1504, 6020, 1591), (3) *P. robustus* (TM 1517, SKX 10924), (4) *H. erectus* (WT 15000, ER 1808, SKX 34805). A model matrix based on these four groups is significantly correlated with the observed Procrustes distance matrix, regardless of whether the four groups are treated as independent (Test #13: *R* = 0.392, *P* = 0.0002) or the two *Paranthropus* groups are coded as linked (Test #14: *R* = 0.504, *P* = 0.0002). On the other hand, if the two *Homo* groups are linked in the model matrix, the correlation drops considerably and the null hypothesis is accepted (Test #15: *R* = 0.104, *P* = 0.153). The above results are the same when OH 62 is excluded from the analyses. For all but two comparisons, morphometric distances (*D_p_*) between members of the two *Homo* groups are significantly high relative to variation in at least one (and usually more than one) extant species (Table 3). Specimens belonging to the two intermediate *Paranthropus* groups generally do not produce significantly high Procrustes distances when paired with those assigned to either form of *Homo* (with some exceptions; SKX 34805 and OH 80, SKX 34805 and ER 6020, ER 6020 and SKX 19495).

**Table 5:** Species attributions for 2-1 Ma hominin humeri based on craniodental association and analysis of distal humeral diaphyseal cross-sectional shape.

Species	*Based on craniodental remains*	*Based on humeral morphology*
*P. robustus*	TM 1517	SKX 10924
*P. boisei*	OH 80	KNM-ER 739, ER 1504, ER 6020, ER 1591
*H. habilis* ^a^	OH 62, KNM-ER 3735	SK 24600, SKX 19495, SK 2598
*H. erectus*	KNM-WT 15000, KNM ER 1808	SKX 34805

**Notes.**

a This group of eastern and southern African fossils is more conservatively referred to as “non-*erectus* early *Homo*” throughout the text, for reasons explained therein.

**Figure 6:**
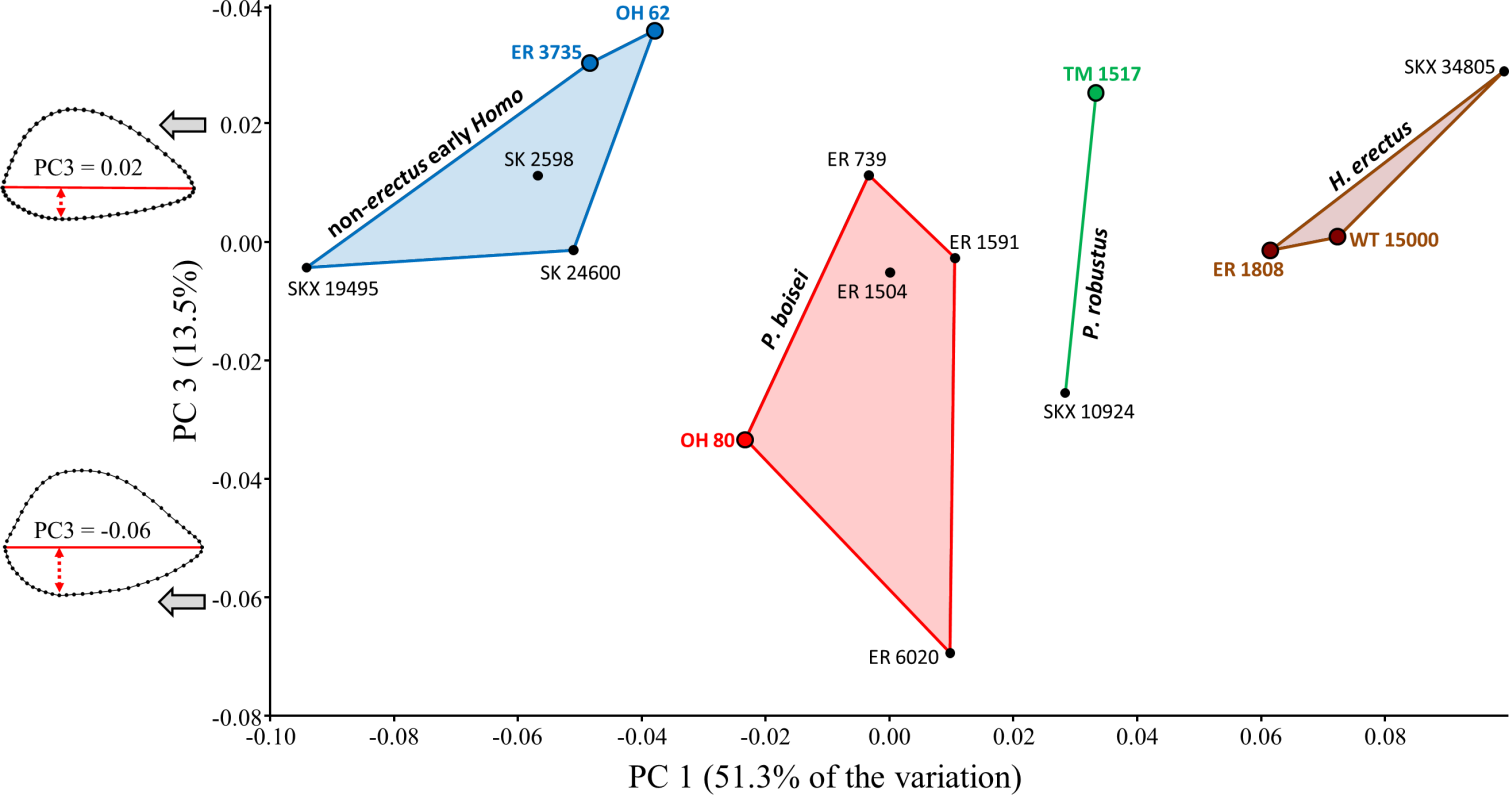
PCA results (PC1, PC3) for the main study group (combined geographic regions). Although groups overlap along PC3, both forms of early *Homo* generally differ from *P. boisei* (on average) by virtue of reduced surface convexity on the posterior side of the diaphysis.

Additional between-group variation is observed along the third principal component (Fig. 6), which is associated with shape differences that (on average) differentiate both forms of early *Homo* from *P. boisei*. Although groups overlap, the two forms of early *Homo* generally exhibit a less convex posterior diaphyseal surface, more similar (but not identical) to the flatter surface observed on the humeri of modern humans. (If the relatively extreme ER 6020 humerus is removed, the same group configuration is observed at a higher principal component (PC5) due to reduced variation along this particular axis).

### Effect of section location on cross-sectional shape variation

The potential effect of variation in section placement was considered by collecting data from four length-defined sections (15%, 17%, 19%, 21%) among modern humans, including the particular section (19%) assumed to be examined on the fossils. On average, among the modern humans sampled here, the proximodistal distance between adjacent sections equals 6.2 mm, such that the most proximal (21%) and most distal (15%) sections are separated by 18.6 mm on a specimen of average humeral length. Hence, the sampled sections cover a relatively wide span of the distal diaphysis.

**Figure 7:**
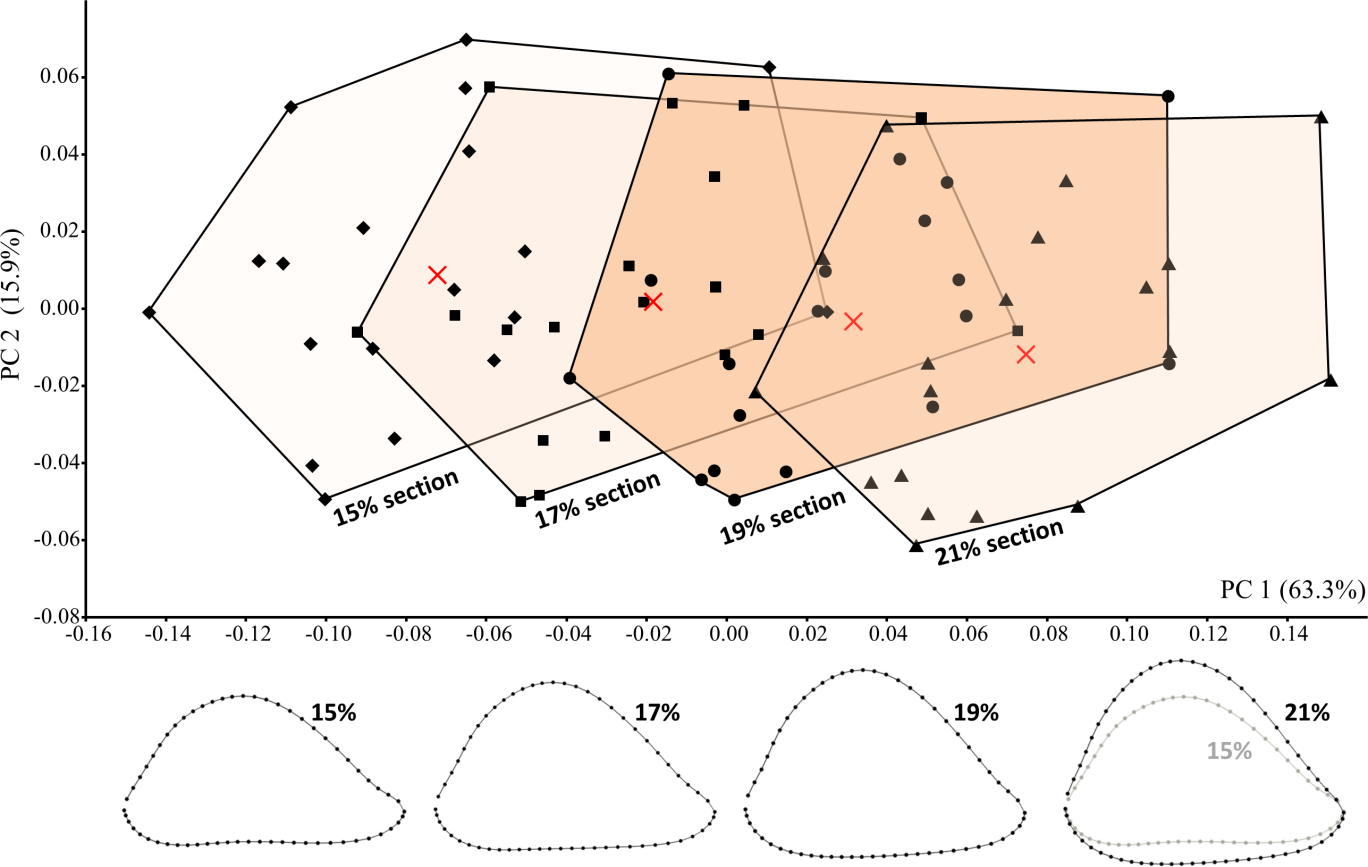
PCA results for modern humans sampled at four different section locations (i.e., 15%, 17%, 19%, and 21% of humeral length). Average landmark configurations are shown for each group (and marked on the plot with “X”). Shape variation along PC1 corresponds to variation in section placement and is associated with increasing AP flatness at more distal sections.

**Figure 8:**
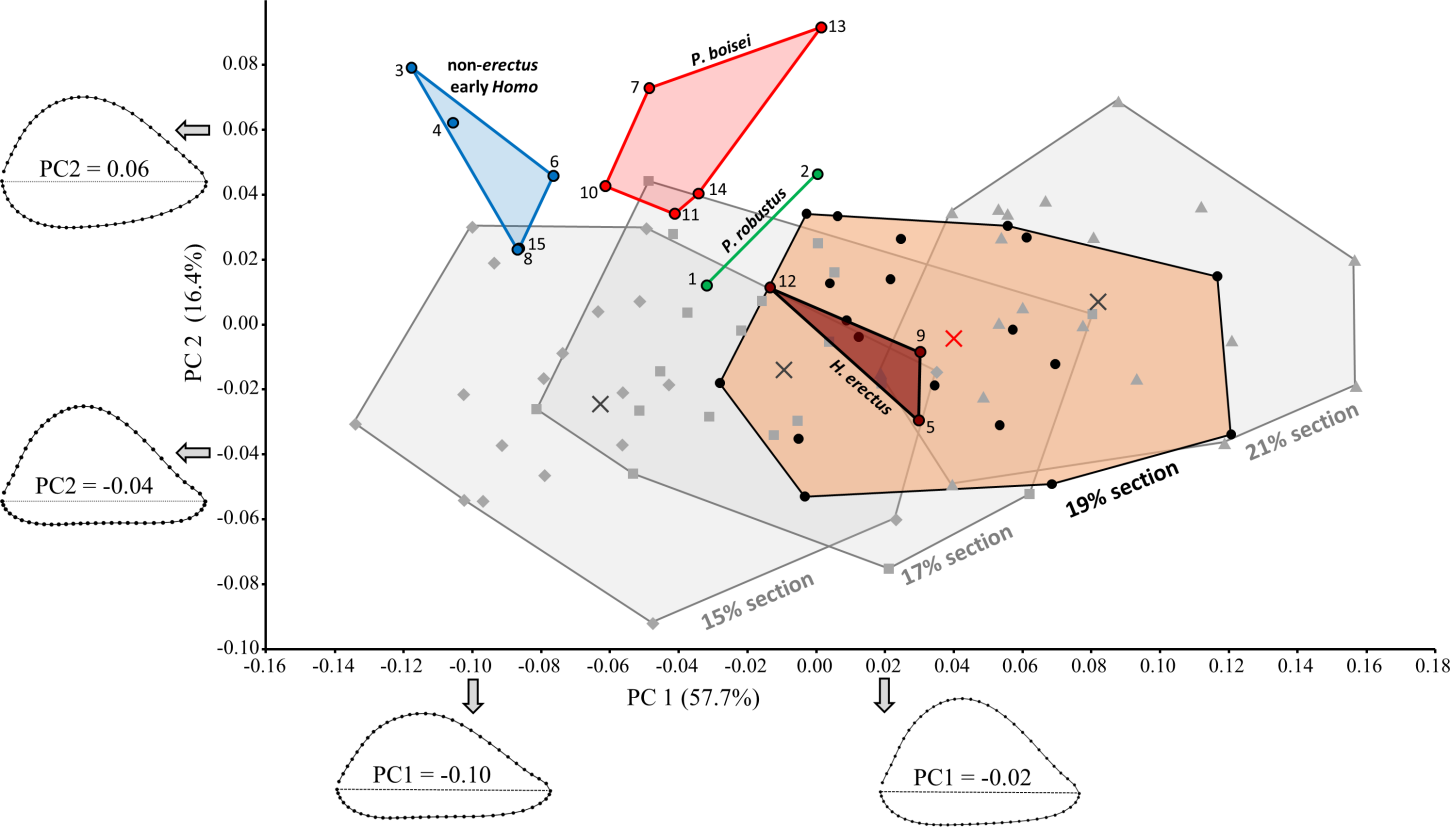
PCA results for the main fossil study group combined with modern humans (at four different section locations). Fossil shapes were quantified at the 19% section level (highlighted for modern humans). More highly AP flattened diaphyseal sections have lower scores along PC1. Even at more distal (i.e., AP flatter) sections, modern humans are dissimilar from the most highly AP flattened fossils (*P. boisei*, non-*erectus* early *Homo*). *H. erectus* fossils resemble modern humans sampled at the same section level (19%). Fossils numbered as in Table 1.

Cross-sectional shape variation related to section placement is largely captured by PC1 (Fig. 7). Not surprisingly, modern human humeral diaphyses becomes relatively flatter in the AP dimension as one moves distally towards the olecranon fossa. In general, only at the most distal (15%) section does the shallow depression just proximal to the olecranon fossa begin to become apparent. Addition of the fossils to the PCA (Fig. 8) slightly changes the placement of PC1, though most of the variation among modern humans (related to section placement) is still associated with this component. The high degree of AP flattening that characterizes *P. boisei* and non-*erectus* early *Homo* is comparable to that observed at more distal regions (15%, 17%) of the modern human diaphysis. Nonetheless, as is clear from PC2, cross-sectional shapes of these two fossils groups (and *P. robustus*) are clearly dissimilar from those observed among modern humans, even at these more distal (i.e., AP flatter) section locations. Variation along PC2 reflects the more oval shape of the non-*erectus* fossil hominins, in contrast to the more triangular shape (and flatter posterior surface) of *H. sapiens*. *H. erectus* is similar to modern humans (particularly the 17% and 19% sections) along both of the first two principal components.

### Comparative context: extant hominids

**Figure 9:**
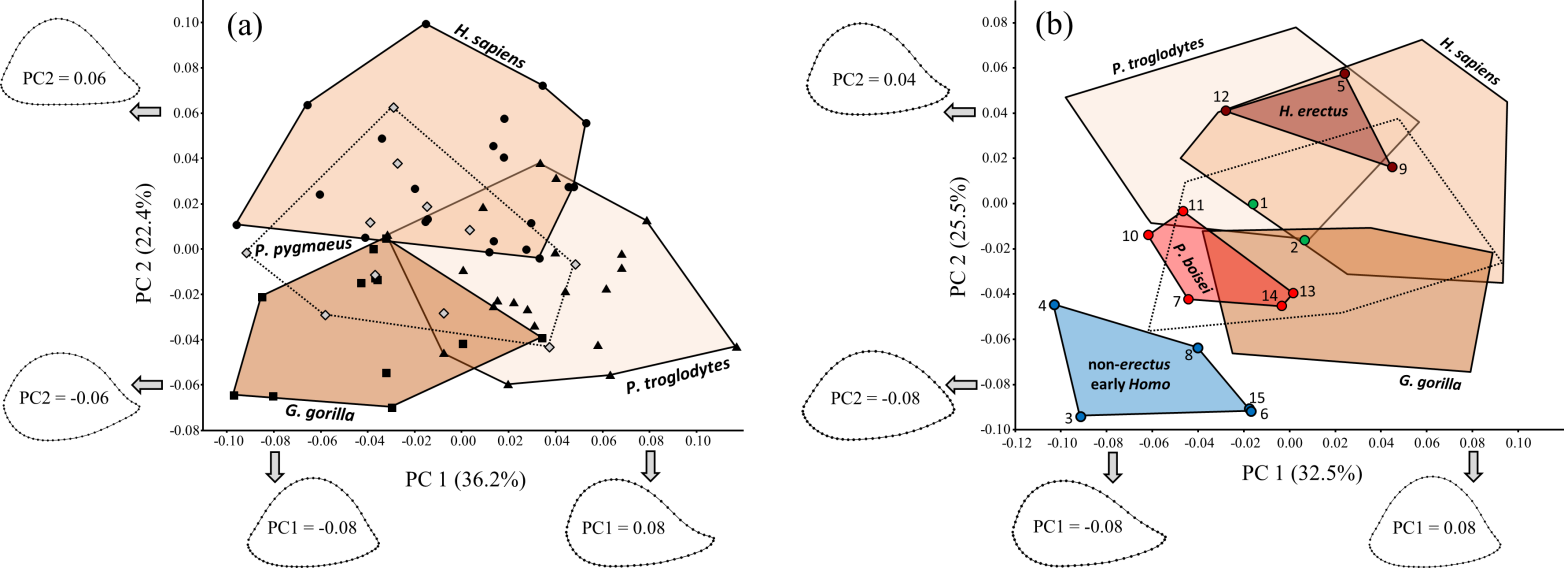
PCA results for (A) extant hominids only, and (B) extant hominids combined with the main fossil study group. Despite considerable overlap, the extant species are significantly different from one another (based on NPMANOVA). Fossils attributed to non-*erectus* early *Homo* are highly unusual among both extant hominids and fossil hominins, while *H. erectus* falls within the *H. sapiens* range of variation. Fossils numbered as in Table 1.

**Figure 10:**
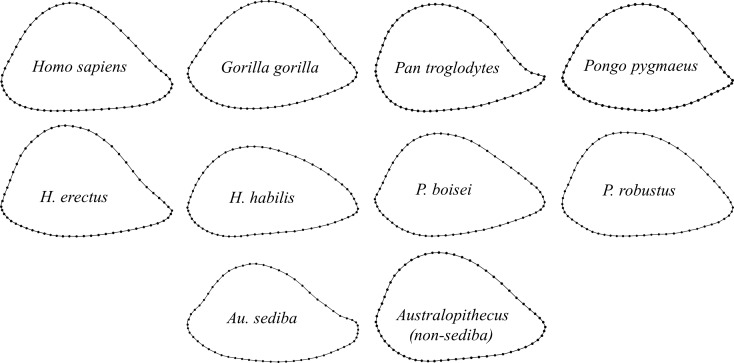
Group averages for distal humeral diaphyseal cross-sectional shape. Shapes are based on generalized orthogonal least-squares Procrustes analysis (GPA) of 60 two-dimensional coordinate points (2 landmarks, 58 sliding semilandmarks).

Non-parametric multivariate analysis of variance (NPMANOVA) based on Procrustes distances (involving random permutation of group membership; 5,000 iterations) indicates significant shape differences among the four extant hominid species (*F* = 10.46, *P* = 0.0002). Based on *post-hoc* tests, all of the extant groups differ significantly from one another in shape (*P* < 0.05), even when the Bonferroni correction is used to adjust *P* values (based on the number of comparisons). Based on PCA of extant hominids alone (Fig. 9A), modern humans differ from nonhuman hominids (particularly African apes) in having a flatter (less convex) posterior diaphyseal surface, resulting in an overall more triangular shape. The lateral edge of the more oval cross-sectional outline of apes is also influenced by a more sharply defined lateral supracondylar ridge, though based on PC1, this is more true for chimpanzees than for the other nonhuman hominid groups (see also Fig. 10). Although orangutans appear to overlap substantially with the other hominids, the first two axes account for only 58.6% of the total variation in the sample; in fact, multiple two-group PCAs involving *Pongo* reveal more substantial differences between orangutans and the other extant species that are obfuscated when all four groups are considered together.

Inclusion of the fossils with the extant taxa (Fig. 9B) produces a similar (albeit rotated) configuration of extant groups. Variation along PC1 is similar to that for the extant-only plot (except that the axis is reversed). The arrangement of the four proposed fossil groups resembles that observed on previous fossil-only PCA plots, such that the two *Paranthropus* species appear intermediate between two different *Homo* extremes. The *H. erectus* group falls within the *H. sapiens* range of variation on PC1 and PC2, while *P. boisei* and non-*erectus* early *Homo* both fall beyond modern humans and do not strongly resemble any particular extant taxon. Indeed, members of the non-*erectus Homo* group appear to be highly unusual among both living and fossil hominids.

### Comparative context: *Australopithecus*

**Figure 11:**
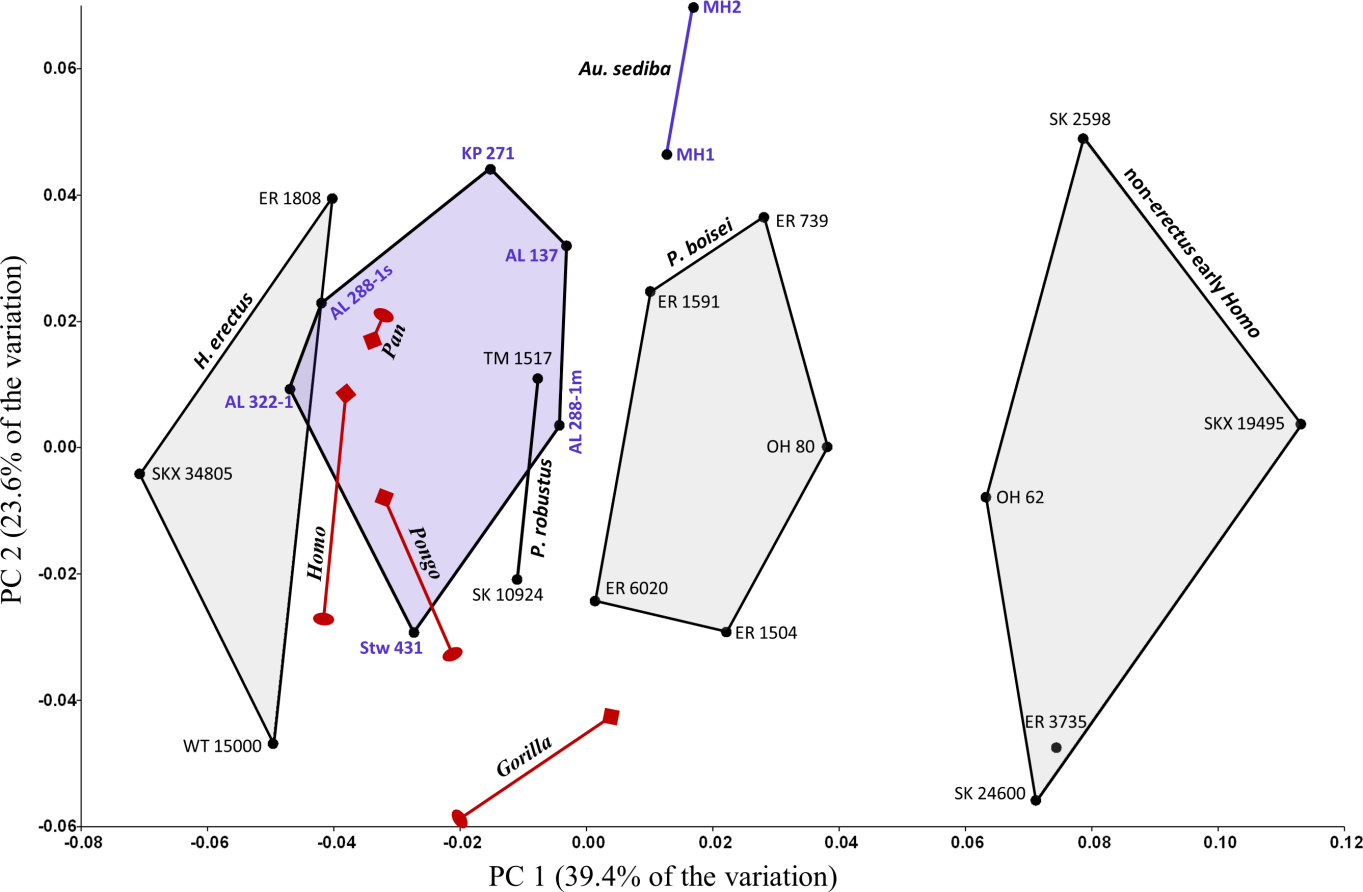
PCA results for the entire fossil sample. Fossils beyond the main study group include seven specimens of *Australopithecus* (purple). Extant hominid sex-species means are also included (dark red; oval, female; diamond, male). Addition of the aforementioned specimens produces similar principal components as a PCA based on the main study group alone. With the exception of those attributed to *Au. sediba*, specimens of *Australopithecus* are relatively similar to one another and to *P. robustus*.

A PCA that includes *Australopithecus* (and sex-species means for extant hominids) yields virtually the same principal components as that for the main study group alone (PC1 reversed), with some additional variation along PC2 due to the high scores for *Au. sediba* (MH1, MH2) along this axis (Fig. 11). Apart from *Au. sediba*, fossil specimens attributed to *Australopithecus* are relatively homogeneous in shape and occupy a region between the proposed *H. erectus* and *P. boisei* groups, thereby overlapping with *P. robustus*.

Although they are clearly not identical in shape, the left and right humeri of AL 288-1 (“Lucy”) are not as relatively dissimilar as they appear along PC1; out of 253 possible pairwise comparisons among the fossil specimens, only six pairings (e.g., MH1 and MH2) yield a lower *D_p_* than that between AL 288-1m and AL 288-1s. Based on Procrustes distances, Lucy’s humeri are more similar to one another than either is to any other fossil specimen, with the result that they are linked together if one performs a UPGMA cluster analysis of the entire fossil sample (not shown, due to an insufficiently high cophenetic correlation coefficient of 0.72). It would appear that the morphological differences between AL 288-1m and AL 288-1s, however small, parallel the shape variation correlated with PC1, accounting for the apparent distance between these specimens along this axis. Indeed, PCA of the *Australopithecus* specimens alone produces a plot in which the Lucy humeri appear (as expected) much closer to one another. Exploration of the extant hominids sampled here does not support the existence of any consistent shape differences between left and right sides, though a thorough analysis of left/right cross-sectional shape asymmetry is beyond the scope of this paper.

**Figure 12:**
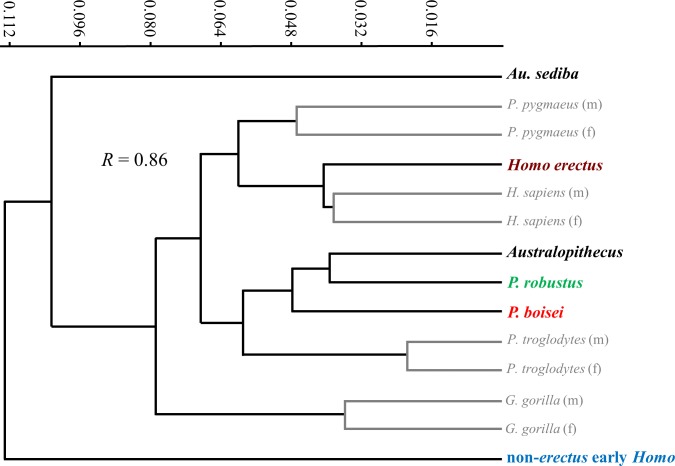
UPGMA cluster analysis based on Procrustes distances between group averages for distal diaphyseal shape. Among extant taxa, all groups are most closely linked to conspecific groups of the opposite sex. “*Australopithecus*” includes all sampled specimens of *Australopithecus* except those attributed to *Au. sediba* and is most closely linked with *P. robustus*. Modern humans are closely linked with *H. erectus*. Both *Au. sediba* and non-*erectus* early *Homo* appear to be unusual among hominids.

Extant species means along PC1 fall close to one another and within the same region as *Australopithecus*, with none approaching the extreme AP flatness observed for specimens of non-*erectus* early *Homo*, and only the average for gorilla males overlapping with *P. boisei*. Based on Procrustes distances, all of the extant species means are about equally dissimilar from the average shape for *Australopithecus* (non-*sediba*), with gorillas being the most distant (as suggested by their higher placement on PC2). The apparent similarity between *Pan* and *Homo* on the PCA plot is somewhat misleading, as chimpanzees are far closer to gorillas than to modern humans along the third principal component (PC3: 14.3%; not shown). In fact, among extant taxa, *Homo* is most similar to *Pongo*, as depicted in a UPGMA cluster analysis based on Procrustes distances between group means (Fig. 12). The uniqueness of both *Au. sediba* and non-*erectus* early *Homo* is also apparent from the cluster analysis, as these groups belong to separate branches that fall outside all of the other sampled hominids. In contrast, *H. erectus* links closely with *H. sapiens*, and *P. robustus* links closely with *Australopithecus* (non-*sediba*). It is also worth noting that all extant hominid sexes are linked with the opposite sex of the same species.

**Figure 13:**
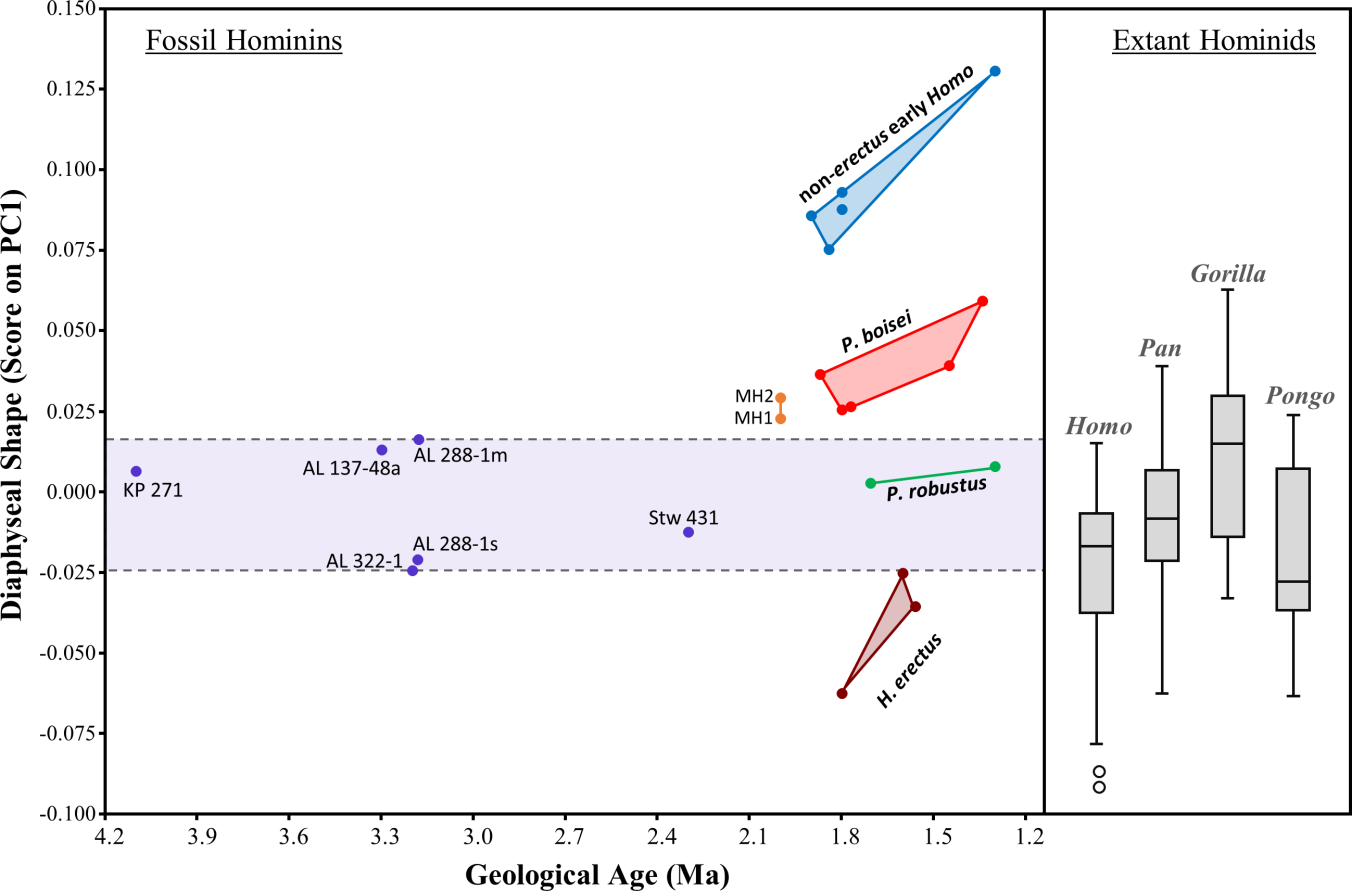
Scatter plot of the relationship between geological time and diaphyseal shape (i.e., PC1 score). PC1 scores were obtained by projecting all specimens onto the first principal component of the combined-region PCA (Fig. 5). Variation among 2-1 Ma fossil hominins is considerable compared to interspecific variation within *Australopithecus*. The relatively AP flattened (ML widened) diaphyses of both *P. boisei* and non-*erectus* early *Homo* are atypical of extant hominids. Boxes for extant species contain 50% of the data (median at horizontal line) and ‘whiskers’ represent min./max. values.

The evolution of humeral shape (at least those aspects captured by PC1) can be further assessed by plotting PC1 scores against geological age of the specimens (Fig. 13). Based on the small sample of specimens included here, the diaphyseal shape of non-*sediba Australopithecus* appears relatively stable over much of the evolution of the genus, particularly considering that the range of PC1 scores observed for *Au. afarensis* includes the scores for both KNM-KP 271 (*Au. anamensis*) and Stw 431 (*Au. africanus*). With the exception of AL 288-1m (just beyond the upper range of *H. sapiens*), all of the scores for non-*sediba Australopithecus* fall within the ranges of the four extant species. The period after 2.0 Ma is characterized by increased variation in humeral diaphyseal shape. While *P. robustus* is characterized by the same morphology exhibited by *Australopithecus*, *H. erectus* exhibits a morphology more similar to modern *Homo* and *Pongo* (also see UPGMA cluster results; Fig. 12). Specimens attributed here to *P. boisei* exhibit a degree of AP flattening that is rarely observed among modern hominids at this level of the diaphysis, while the diaphyseal shape of non-*erectus* early *Homo* appears to be unique among all of the extant and fossil hominids sampled here.

**Figure 14:**
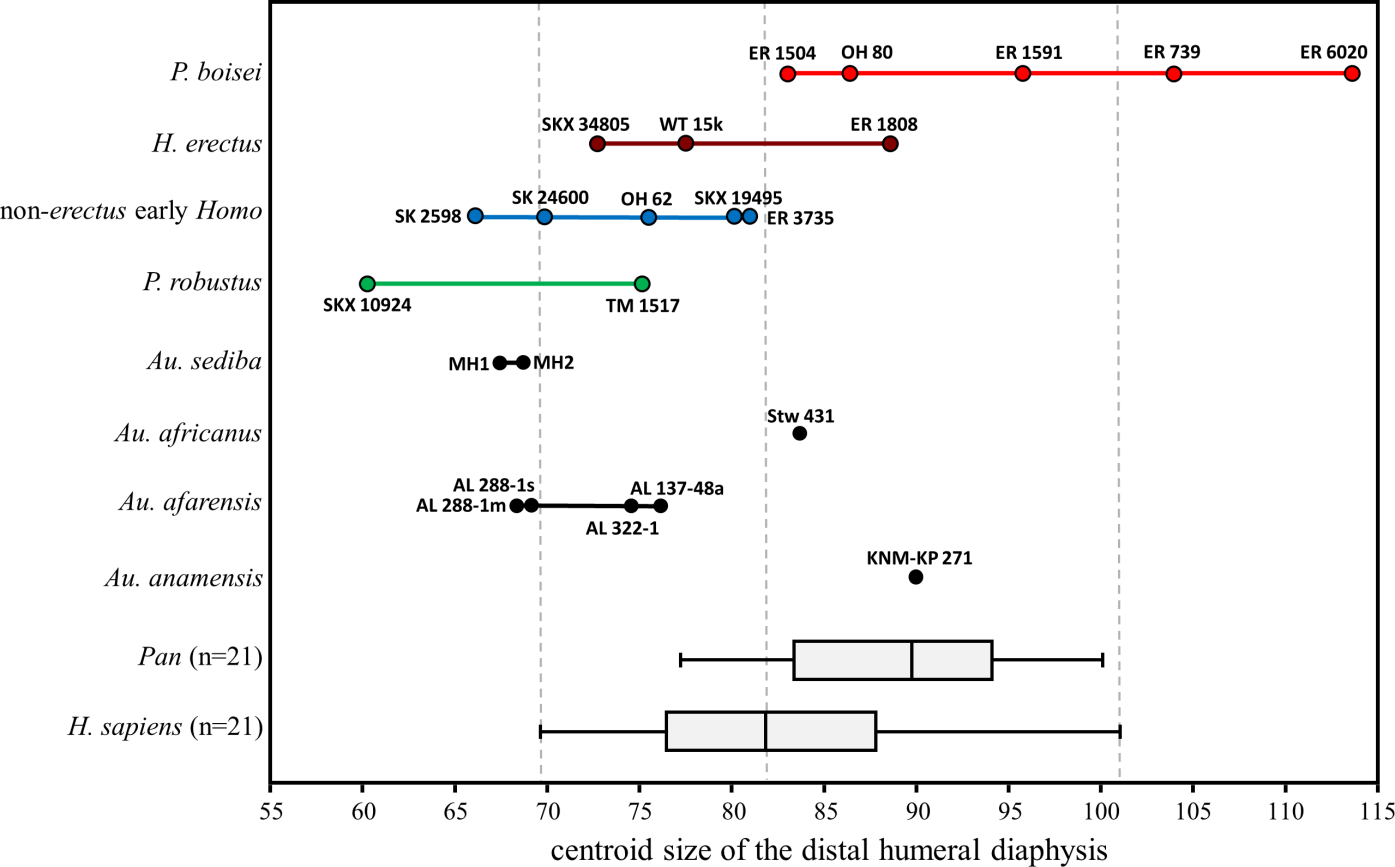
Cross-sectional size of the distal humeral diaphysis (base on centroid size). Boxes for extant species contain 50% of the data (median at horizontal line) and ‘whiskers’ represent min./max. values. *Gorilla* and *Pongo* not shown due to their extreme size.

With respect to overall size of the cross section, the majority of fossils fall in the lower half of the *H. sapiens* range (Fig. 14). Exceptions among the *Australopithecus* specimens include the moderately large *Au. africanus* humerus (Stw 431) and the somewhat larger *Au. anamensis* humerus from Kanapoi (KNM-KP 271), both of which fall in the upper size range for modern humans. Most of the fossil *Homo* specimens are similar in size to those of smaller-bodied hominins, with the exception of KNM-ER 1808 (*H. erectus*). The size overlap between *H. erectus* and non-*erectus* early *Homo* indicates that the substantial shape differences between these groups are not allometric in nature. Within *P. boisei*, even the three smallest specimens fall in the upper half of the modern human size range, while the two largest specimens are beyond the range of modern humans and chimpanzees sampled here (and similar in size to small gorillas and large orangutans; not shown).

## Discussion

In both southern and eastern Africa, the distal humeral diaphysis is useful for differentiating among Lower Pleistocene hominin taxa. In each region, diaphyseal shape variation indicates at least three distinct morphotypes (*Paranthropus*, *Homo erectus*, non-*erectus* early *Homo*) during the time period under study (c. 2-1 Ma), and does not reflect a simple dichotomy between *Paranthropus* and *Homo*. While the two species of *Paranthropus* are somewhat similar with respect to diaphyseal shape, the two *Homo* groups are distinctly different from one another, reflecting the presence of at least two postcranial morphotypes within early *Homo* (Ward et al., 2015). Overall, *H. erectus* exhibits a diaphyseal profile similar to modern humans, while specimens attributed here to non-*erectus* early *Homo* are characterized by a high degree of anteroposterior flattening (relative to mediolateral width) that is unusual among hominids. Shape differences between the two *Homo* groups are also apparent along the anterolateral surface, which is slightly concave in *H. erectus*, but slightly convex in non-*erectus* early *Homo*. Although *P. boisei* humeri are also unusually AP flattened, they exhibit a morphology that is intermediate in many ways between the two *Homo* extremes. On balance, the nine craniodentally unassociated humeral specimens examined here are best attributed as follows (also see Table 5): (1) *Homo aff. habilis* (SK 24600, SKX 19495, SK 2598), (2) *Homo erectus* (SKX 34805), (3) *Paranthropus boisei* (ER 739, 1504, 6020, 1591), (4) *Paranthropus robustus* (SKX 10924).

### South Africa

The results of this study lend credence to previous claims that diaphyseal morphology can be used to identify early hominin humeri at Swartkrans (Susman, de Ruiter & Brain, 2001). Nonetheless, these results also support suggestions (based on elbow morphology; Lague, 2014) that the original taxonomic attributions of the Swartkrans humeri should be revised. Variation in diaphyseal morphology at Swartkrans suggests the presence of at least two species of early *Homo*, one with affinities to eastern African *Homo habilis* (“1813 group”; SK 24600, SK 19495, SK 2598), and one with affinities to *Homo erectus* (SKX 34805). Moreover, both distal articular and diaphyseal cross-sectional morphology indicate that SKX 10924 is best attributed to *P. robustus*, as it bears the greatest resemblance to TM 1517 (*P. robustus* holotype) in both anatomical regions. Although Susman, de Ruiter & Brain (2001) allocated SKX 34805 to *Homo* (in agreement with the present study), this suggestion was based on the assumption that *all* of the more rounded specimens from Swartkrans (including SKX 10924) represent *Homo*; this does not appear to be the case.

Two of the aforementioned Swartkrans humeri (SKX 10924, SKX 19495) derive from Member 3. Hence, although the particular species attributions proposed here for these specimens differ from previous attributions, the implications are the same: while none of the craniodental specimens from Member 3 have been attributed to *Homo* (Grine et al., 2009), the humeral evidence indicates that both *Homo* (at least one specimen) and *Paranthropus* are present in these deposits. The Member 3 deposits may therefore contain one of the latest-occurring examples of non-*erectus* early *Homo* in Africa (i.e., SKX 19495), depending upon the appropriate dating of this member (e.g., Balter et al., 2008; Herries, Curnoe & Adams, 2009; Sutton et al., 2009).

This study finds no morphological basis for differentiating the South African form of non-*erectus* early *Homo* from contemporaneous *H. habilis* in eastern Africa. Nevertheless, it should not be automatically assumed that the *H. habilis* specimens (ER 3735, OH 62) are conspecific with the similarly flattened Swartkrans specimens (SK 24600, SKX 19495, SK 2598). Studies based on craniodental material suggest that South African early *Homo* fossils may represent species (or a species lineage) not sampled in eastern Africa (Grine et al., 1993; Grine et al., 2009); this hypothesis is not refuted by the present results, particularly given that relatively homogeneous humeral morphology can be observed across multiple species of *Australopithecus* for both the elbow joint (Lague & Jungers, 1996; Lague, 2014) and the distal diaphysis. Hence, it is plausible that different species of non-*erectus* early *Homo* may also share similar humeral morphology. At this time, it is reasonable to refer to the South African members of the “non-*erectus* early *Homo*” group as *H. aff. habilis*.

### Eastern Africa

In eastern Africa, the enigmatic humeri from Koobi Fora (KNM-ER 739, 1504, 6020, 1591) are best attributed to *Paranthropus boisei*, as has long been suspected for both ER 739 and ER 1504 since their original attributions to *Australopithecus* (Leakey, Mungai & Walker, 1972; Leakey, 1973). It is not particularly surprising that these specimens share similar diaphyseal morphology, since multiple studies have noted strong similarities among ER 739, 1504 and 6020 with respect to elbow joint morphology (Leakey, 1973; Senut, 1981c; Lague & Jungers, 1996; Lague, 2014; but see McHenry & Brown, 2008). KNM-ER 1591 has received less attention than the other Koobi Fora specimens, probably because it lacks most of its articular anatomy. Although it was originally attributed to *Homo* (Leakey, 1973), its status as a hominin has been questioned (Walker & Leakey, 1993); in fact, it has recently been listed in the Turkana Public Database as a carnivoran. Nevertheless, it does not resemble the humerus any known extant or fossil carnivoran (ME Lewis, pers.comm., 2015) and its contours are remarkably similar to those of KNM-ER 739 throughout the length of the diaphysis (most of which is preserved). Hence, KNM-ER 1591 and KNM-ER 739 are likely conspecific.

With respect to its distal diaphysis, the OH 80 *P. boisei* humerus was originally described as AP flat (ML broad) and therefore similar in shape to the “*P. robustus*” specimens of Susman, de Ruiter & Brain (2001; i.e., SK 2598, SK 24600, SKX 19495). Based on the present study, however, the specimens attributed by Susman and colleagues to *P. robustus* actually represent a form of non-*erectus* early *Homo*. In fact, Domínguez-Rodrigo et al. (2013) also note that the OH 80 humerus has a relatively *greater* AP dimension than the presumed “*P. robustus*” specimens. Although Domínguez-Rodrigo et al. (2013) surmise that this discrepancy might be due to sex differences (or comparisons of slightly different sections), their observations accord well with the results of the present study. Although OH 80 is relatively AP flattened compared to *H. erectus* and modern humans, it does, in fact, have a relatively greater AP dimension than humeri attributed here to non-*erectus* early *Homo* (including the “*P. robustus*” specimens of Susman, de Ruiter & Brain, 2001).

The intermediate degree of AP flattening observed for *P. boisei* is accompanied by a high degree of posterior surface convexity (particularly medially) that is apparent upon visual inspection (contrasting with the flatter posterior surface generally observed in *Homo*). The diaphyseal morphology of *P. boisei* noted here augments a list of anatomical features that render the humeri of this species fairly distinctive, including highly projecting lateral and medial epicondyles, a mediolaterally narrow zona conoidea, wide medial and lateral pillars, and a shallow olecranon fossa (Leakey, 1973; Senut, 1981c; Lague & Jungers, 1996). In addition, it appears that *P. boisei* had unusually large arms compared to both earlier and contemporaneous hominins, with the larger specimens exhibiting features either at the upper end (elbow joint; Lague, 2014) or beyond (distal humeral diaphysis) the size range of modern humans. Moreover, the OH 80 humerus is characterized by cortical bone that is considerably thicker than that of modern *H. sapiens* (Domínguez-Rodrigo et al., 2013), and the well-preserved diaphyses of both KNM-ER 739 and ER 1591 have extremely well-pronounced muscle markings. The body size range of *P. boisei* remains speculative, though it is clear from a number of specimens (i.e., KNM-ER 739, ER 6020, ER 1591, OH 80) that this species was characterized by extremely robust, powerful upper limbs. It is unknown whether the humeral strength of *P. boisei* is high relative to femoral strength (as demonstrated for *H. habilis*; Ruff, 2009), though the powerful upper limbs of *P. boisei* are consistent with frequent arboreal climbing.

As surmised by Lague & Jungers (1996), it is theoretically possible that one or more of the craniodentally unassociated Koobi Fora humeri represents *H. rudolfensis* (i.e., the “1470 group”; Antón, Potts & Aiello, 2014; Spoor et al., 2015) rather than *P. boisei*. Nonetheless, fossils attributed to *H. rudolfensis* are rare above the KBS Tuff (Antón, Potts & Aiello, 2014; Joordens et al., 2013), and other than KNM-ER 3735 (*H. habilis*), KNM-ER 1504 is the only Turkana specimen examined here that derives from below this level. Moreover, the strong similarity among the Koobi Fora humeri and the distinctive nature of both their articular and diaphyseal morphology suggests that they all belong to the same genus, if not the same species. Given that KNM-ER 739 is a good deal younger (c. 1.5 Ma) than any currently known *H. rudolfensis*, and that all of the Koobi Fora specimens resemble OH 80 with respect to diaphyseal shape, it is most likely that these specimens solely represent *P. boisei*. An alternative (but less likely) possibility is that, in contrast to all other known forms of early *Homo* (*H. habilis*, *H. erectus*, South African non-*erectus Homo*), the humeri of *H. rudolfensis* are indistinguishable from those of *P. boisei*. Further light on this issue awaits the discovery of postcranial remains that can be definitively linked to *H. rudolfensis* by virtue of craniodental association.

### Additional implications of this study

The work presented here solves a small piece of the postcranial puzzle that has long impeded progress in reconstructing the evolution and functional morphology of Lower Pleistocene hominins. As originally suggested by the work of Senut (1981a), humeral diaphyseal morphology can facilitate the taxonomic identification of specimens lacking associated diagnostic craniodental remains.

The results presented here have implications that extend beyond the particular set of fossil humeri under examination. For example, AP diaphyseal flattening has previously been observed for KNM-ER 739 in comparison to Gomboré IB 7594^4^4Unfortunately, this specimen was unavailable for this study. (Melka Kunturé; Chavaillon et al., 1977; Senut, 1981a). Given the nature of diaphyseal variation observed here, the Melka Kunturé humerus, originally attributed to *Homo sp*. (Chavaillon et al., 1977), therefore most likely represents *H. erectus*. In addition, two of the craniodentally unassociated humeri examined here are associated with additional postcranial elements. The SK 24600 humerus is thought to belong to the same individual as a proximal radius (SK 24601; Susman, de Ruiter & Brain, 2001). The results presented here therefore imply that the morphology of the SK 24601 radius is characteristic of non-*erectus* early *Homo* (a proposition that merits further testing via morphometric comparisons with other available proximal radii; e.g., OH 80-11, OH 62, KNM-ER 3735, KNM-ER 1500, SK 18b, SKX 3699). In addition, the KNM-ER 1504 humerus (herein attributed to *P. boisei*) is associated with two partial femora (ER 1503, ER 1505) thought to represent the same individual (Leakey, 1973). The ER 1503 proximal femur bears a primitive morphology common among australopiths (and *Orrorin tugenensis*), such as a small femoral head, a long and anteroposteriorly narrow femoral neck, low neck-shaft angle, and broad proximal shaft (Richmond & Jungers, 2008; Harmon, 2009; Richmond & Jungers, 2012). Hence, the results of the present study confirm that typical australopith femoral morphology also characterized *P. boisei*.

Distal humeral diaphyseal shape variation among Lower Pleistocene hominins does not resemble variation observed among extant great apes and humans, making it difficult to draw functional inferences based on comparative anatomy. The similarities in diaphyseal anatomy between *Homo* and *Pongo* are particularly intriguing given obvious differences in habitual use of the upper limb. Although long bone cross-sectional properties are known to be responsive to mechanical loadings during life (Ruff, Holt & Trinkaus, 2006; Ruff, 2009), the extent to which distal humeral diaphyseal shape responds to habitual limb use (or is phylogenetically constrained) is unclear. Strongly marked entheseal changes associated with muscles that cross the shoulder have been correlated with differences in humeral diaphyseal shape (and diaphyseal rigidity) in the mid-proximal region, though a similar phenomenon has not been observed in the distal diaphysis (Ibáñez-Gimeno et al., 2013). It may be the case that distal diaphyseal shape is largely unaffected by age-related or activity-related variation in cross-sectional properties. Although relative cortical thickness of the mid-distal shaft of the KNM-WT 15000 humerus is much lower than that of KNM-ER 1808T (likely due to its subadult status; Ruff, 2008), the two conspecific specimens are very similar in distal diaphyseal shape. In fact, the same can also be said for MH1 and MH2, which also represent conspecific subadult and adult individuals, respectively. Hence, limited evidence would suggest that, at least by a certain age, skeletally immature individuals have a similar distal diaphyseal shape to that of adults, even if differences exist in relative cortical thickness (and therefore diaphyseal strength).

The shape of the diaphyseal section sampled here has the potential to be affected by the salience of the lateral supracondylar ridge, which could influence total mediolateral width as well as the lateral profile of the section (as is particularly the case for chimpanzees). On the other hand, the mediolaterally widest fossil specimens in this study (non-*erectus* early *Homo*) have fairly rounded lateral borders and are not characterized by a strongly developed lateral ridge, suggesting that variation in AP/ML width among fossil hominins is not simply being driven by variation in the lateral projection of this feature.

It is plausible that distal diaphyseal shape variation is at least partly related to different patterns of force transmission through the distal region of the humerus. The typical location of maximum transarticular forces would be expected to affect the placement of the anteroposteriorly thickest region of the distal diaphysis (which, among hominids, is typically located proximal to some part of the trochlea). In this regard, it is reasonable to surmise that aspects of articular morphology will be correlated with aspects of distal diaphyseal shape, since both should reflect typical forces through the elbow. For example, greater force through the medial side of the humeroulnar joint (perhaps due to powerful musculature arising from the medial epicondyle) might necessitate both an increase in the surface area of the medial trochlea (and therefore the size of medial trochlear crest), and the addition of bone on the medial pillar, thereby increasing relative mediolateral width of the diaphysis. Future investigations will explore the functional relationships between joint morphology, diaphyseal shape, and the power/leverage of forearm muscles originating from the medial and lateral epicondyles.

Although incorporation of additional fossil humeri may expand the variation observed within *Australopithecus*, present evidence indicates that the diaphyseal shape of this genus is characterized by a pattern of long-term stability. Among species of *Australopithecus*, only the late-occurring *Au. sediba* exhibits morphology that is unusual (and unique among hominids). In addition, the distal diaphyseal morphology of *P. robustus* resembles that of non-*sediba Australopithecus*. A pattern of long-term postcranial homogeneity among australopiths is not unique to the humeral diaphysis, as it has also been observed for elbow joint morphology (Lague & Jungers, 1996; Lague, 2014) and for proximal femoral morphology (Richmond & Jungers, 2008; Richmond & Jungers, 2012). It is worth noting, however, that although the proximal femur of *P. boisei* does not appear to appreciably differ from that of other australopiths (based on KNM-ER 1504), the distal humerus of *P. boisei* departs from the general australopith pattern for both joint morphology and diaphyseal cross-sectional shape.

With respect to distal humeral diaphyseal morphology, the general similarities observed among *Australopithecus* (non-*sediba*), *P. robustus*, and multiple extant hominids suggest that non-*sediba Australopithecus* morphology represents a primitive condition relative to the other fossil hominins. The marked variation in diaphyseal morphology observed among post-*Australopithecus* hominin species may reflect an increased diversity of forelimb use in the Lower Pleistocene, perhaps associated with the more pronounced habitat/resource heterogeneity associated with this time period (Antón, Potts & Aiello, 2014). The unique diaphyseal morphology associated with non-*erectus* early *Homo* presents a particularly striking contrast to the more modern-looking diaphysis of *H. erectus*, further emphasizing observations of postcranial diversity within early *Homo* (e.g., Ruff, 2009; Ward et al., 2015), as well as previous descriptions of early *Homo* diversification as a time of “morphological experimentation” (Antón, Potts & Aiello, 2014). Among the fossil species examined here, *H. erectus* shows the greatest similarities to *H. sapiens*, perhaps indicating a shift to a more modern pattern of upper limb use compared with earlier and contemporaneous hominin species (i.e., increased manipulatory skill in a fully committed terrestrial biped). The unique morphology of *Homo habilis* is more difficult to interpret without further investigation , though this species may be unusual among early hominins in the use of its upper limbs for *both* primitive stone tool production and (according to some researchers) habitual arboreal climbing behavior. A more complete understanding of upper limb function in early hominin taxa awaits the results of ongoing research.

## Conclusions

In both southern and eastern Africa, the distal humeral diaphysis is useful for differentiating among early (c. 2-1 Ma) hominin taxa (i.e., *Paranthropus*, *H. erectus*, non-*erectus* early *Homo*). While previous claims that diaphyseal morphology can be used to identify taxa at Swartkrans (Susman, de Ruiter & Brain, 2001) are supported, the original taxonomic attributions of the Swartkrans humeri should be revised (as is also suggested by distal articular anatomy; Lague, 2014). For example, those specimens previously referred to *P. robustus* (SK 2598, SKX 19495, SK 24600) should be allocated to a form of non-*erectus* early *Homo* (e.g., *H. aff. habilis*), while evidence from both the elbow joint and the distal diaphysis indicates that SKX 10924 belongs to *P. robustus*. The enigmatic isolated humeri from Koobi Fora (KNM-ER 739, 1504, 6020, 1591) all resemble OH 80 and are best attributed to *P. boisei*. Overall, the diaphysis of both *P. boisei* and non-*erectus* early *Homo* is unusually AP flattened (ML broad) among both fossil hominins and extant hominids, though non-*erectus Homo* humeri are most extreme in this regard and markedly different from the more modern human-like specimens attributed to *H. erectus*.
